# Effects of Rice Bran Supplementation on Metabolic Syndrome-Related Parameters: A Systematic Review and Meta-Analysis

**DOI:** 10.3390/ijms26189051

**Published:** 2025-09-17

**Authors:** Sirapatsorn Tantayakhom, Ratchanon Inpan, Kantirat Yaja, Nut Koonrungsesomboon, Supanimit Teekachunhatean, Mingkwan Na Takuathung

**Affiliations:** 1 Department of Pharmacology, Faculty of Medicine, Chiang Mai University, Chiang Mai 50200, Thailand; sirapatsorn.tan@gmail.com (S.T.); ratchanon.inpan@gmail.com (R.I.); kantirat.y@gmail.com (K.Y.); nkoonrung@gmail.com (N.K.); supanimit.t@cmu.ac.th (S.T.); 2 Clinical Research Center for Food and Herbal Product Trials and Development (CR-FAH), Faculty of Medicine, Chiang Mai University, Chiang Mai 50200, Thailand; 3 Office of Research Administration, Chiang Mai University, Chiang Mai 50200, Thailand

**Keywords:** rice bran, metabolic syndrome, anthropometric parameters, dyslipidemia, insulin resistance

## Abstract

Rice bran, a fiber-rich source of bioactive compounds, has gained attention for its potential health benefits, yet its effects on metabolic syndrome (MetS) remain unclear. This study aimed to evaluate the impact of rice bran consumption on anthropometric measures, blood pressure, glycemic control, and lipid profiles in individuals with MetS. A systematic search of PubMed/Medline, Scopus, Cochrane Library, and Embase was conducted up to 30 January 2025, to identify randomized controlled trials (RCTs) assessing rice bran supplementation. Twenty-six RCTs involving 1255 participants (642 in rice bran groups, 613 in controls) were included in the meta-analysis. Weighted mean differences (WMDs) with corresponding *p* values were calculated. Rice bran significantly reduced systolic blood pressure (WMD: −3.336 mmHg; *p* = 0.0006), diastolic blood pressure (WMD: −3.145 mmHg; *p* = 0.015), and HbA1c (WMD: −0.199%; *p* = 0.003). Lipid profiles improved, with reductions in total cholesterol (WMD: −13.594 mg/dL; *p* < 0.0001) and LDL cholesterol (WMD: −14.580 mg/dL; *p* < 0.0001), and an increase in HDL cholesterol (WMD: 3.074 mg/dL; *p* = 0.007). These findings suggest rice bran supplementation may be a promising natural dietary strategy for managing and preventing MetS.

## 1. Introduction

Metabolic syndrome (MetS) is a growing global health concern, affecting nearly one-fifth of the population in the United States of America and Europe, with a rising incidence in Southeast Asia [1]. A recent global meta-analysis involving over 28 million participants reported a worldwide prevalence ranging from 12.5% to 31.4%, with the highest rates observed in the Eastern Mediterranean and the Americas. Central obesity and elevated blood pressure were the most prevalent components, highlighting the substantial and rising global burden of MetS [2]. It is a complex disorder and is diagnosed when at least three of the following five conditions are present: (1) abdominal obesity (waist circumference >40 inches in men, >35 inches in women), (2) elevated blood pressure (≥130/85 mmHg), (3) impaired fasting glucose (≥100 mg/dL), (4) fasting triglycerides ≥150 mg/dL, and (5) reduced high-density lipoprotein cholesterol (HDL-C) (<40 mg/dL in men, <50 mg/dL in women) [3]. MetS significantly contributes to the increasing prevalence of cardiovascular disease, type 2 diabetes, and obesity-related complications, and is linked to higher mortality rates and cancer risk due to potential DNA damage [4]. Through persistent cytokine activation, chronic low-grade inflammation—which is frequently seen in MetS—promotes the initiation and spread of tumors [5]. The insulin/IGF-1 axis is stimulated by insulin resistance and compensatory hyperinsulinemia, which promotes cell division and prevents apoptosis [6]. Given its widespread impact, effective prevention and management strategies are needed.

The current management of MetS focuses on addressing the risk factors and targeting the individual components of MetS. Therapeutic approaches aim to regulate anthropometric measurements, blood pressure, glycemic levels, and lipid levels [7]. While pharmacological treatments, such as antihypertensives, lipid-lowering agents, and glucose-lowering agents, are commonly used, they may cause adverse effects, including gastrointestinal disturbances, liver toxicity, and an increased risk of hypoglycemia [7]. Additionally, long-term medication adherence is challenging due to factors such as cost, adverse effects, and compliance issues [8,9]. Consequently, lifestyle modifications play a crucial role in MetS prevention and management. Although comprehensive lifestyle changes typically include both dietary and physical activity components, evidence suggests that dietary interventions alone can significantly improve metabolic parameters and reduce MetS risk independently of physical activity levels. This justifies our focus on dietary intervention in this systematic review. Strategies such as increasing fiber intake and replacing animal fats with vegetable fats are widely recommended for their beneficial effects on lipid metabolism, insulin sensitivity, and inflammation reduction [10,11]. Furthermore, dietary patterns like the Mediterranean one or dietary approaches to stop hypertension, which emphasize whole grains, legumes, fruits, vegetables, and healthy fats, while limiting processed foods, have been shown to significantly improve MetS-related parameters [12,13]. Emerging evidence also suggests that bioactive compounds from functional foods, including polyphenols, flavonoids, and phytosterols, can modulate metabolic pathways, offering additional therapeutic potential for MetS management [14,15].

Rice bran has emerged as a promising functional food for MetS management due to its rich nutritional profile and bioactive compounds. Derived from rice during the milling process, rice bran is abundant in dietary fiber, γ-oryzanol, tocopherols, tocotrienols, and essential fatty acids like oleic and linoleic acids [16]. These bioactive components contribute to its metabolic benefits, including improvements in lipid profiles by lowering total cholesterol, low-density lipoprotein cholesterol (LDL-C), and triglycerides [17,18]. Additionally, rice bran enhances glycemic control by reducing fasting blood glucose (FBG) and glycated hemoglobin (HbA1c) [19], aids in body weight regulation through its high fiber content, and supports blood pressure regulation and endothelial function due to its antioxidant and anti-inflammatory properties [18,19,20]. Given these multifaceted effects, rice bran represents a promising dietary intervention for mitigating MetS-related risk factors and improving metabolic health.

Despite growing interest, clinical evidence on rice bran’s broader metabolic effects remains unclear. Previous systematic reviews and meta-analyses have primarily focused on its lipid-lowering properties [21], leaving significant gaps in understanding its impact on other MetS-related parameters and their potential. This systematic review and meta-analysis aimed to fill these gaps by quantitatively analyzing recent clinical data to assess the effects of rice bran supplementation on key components of MetS, including individual endpoints such as fasting blood glucose, lipid parameters (total cholesterol, LDL-C, HDL-C, and triglycerides), blood pressure (systolic and diastolic), and anthropometric measures (body mass index (BMI) and waist circumference), as well as overall improvements in MetS-related profiles.

## 2. Materials and Methods

The study was conducted following the Preferred Reporting Items for Systematic Reviews and Meta-Analyses (PRISMA) guidelines to ensure rigorous data processing, analysis, and reporting [22]. This study was registered in PROSPERO (CRD42024582960) and was granted an exempt research determination by the Research Ethics Committee of the Faculty of Medicine, Chiang Mai University (EXEMPTION 0544/2024).

### 2.1. Search Strategy

A systematic search of four electronic databases, namely PubMed/Medline, Scopus, Cochrane Library, and Embase, was conducted on 1 January 2024, (with the last update on 30 January 2025) to identify relevant studies. Two reviewers independently performed the literature search to ensure comprehensive coverage and minimize selection bias. The search strategy incorporated key terms related to the intervention (i.e., rice bran, rice bran oil, rice oil, and oryzanol) and the outcome of interest (i.e., body mass index, waist circumference, systolic blood pressure, diastolic blood pressure, fasting blood glucose, HbA1c, insulin, triglycerides, total cholesterol, LDL-C, and HDL-C). These domains were combined using the “AND” operator to refine the search. Duplicate records were identified and removed using EndNote (version 21).

### 2.2. Study Selection and Eligibility Criteria

The screening process began with an initial review of titles and abstracts to identify potentially eligible studies. Two reviewers independently screened titles and abstracts, and any discrepancies were resolved through discussion or consultation with a third reviewer. The inclusion criteria were limited to RCTs evaluating the effects of rice bran, rice bran oil, or its bioactive compound (oryzanol) supplementation on MetS-related markers compared to a control group. Studies were required to have MetS-related parameters. Studies were excluded if they were in vitro or animal experiments, review articles, case reports, expert opinions, conference proceedings, or book chapters. These sources were excluded due to their lack of peer review, insufficient methodological details, and limited suitability for quantitative synthesis. The screening and selection of eligible studies were conducted independently by two authors. Any disagreements that arose during this process were resolved through consultation with a third author.

### 2.3. Data Extraction

The following information was extracted from the included studies: publication details (first author’s name, journal title, year of publication, and study location), participant characteristics (health status, age, and gender), intervention details (type/form, dosage, and duration of intervention), and outcome measures. The outcomes of interest included MetS-related markers, such as anthropometric parameters (e.g., BMI and waist circumference), blood pressure (e.g., systolic blood pressure (SBP) and diastolic blood pressure (DBP)), glycemic parameters (e.g., FBG, HbA1c, and insulin levels), and lipid profiles (e.g., triglycerides, total cholesterol, LDL-C, and HDL-C).

For each outcome, data were extracted to determine the mean change from the baseline and the corresponding standard deviations (SDs). If a study did not directly report the mean change and SD, or if the SD was unavailable, alternative metrics were used. Imputation was performed following the methods recommended by the Cochrane Handbook for Systematic Reviews of Interventions [23]. This included deriving the SD from the standard error (SE) or 95% confidence interval (CI), where applicable. Additionally, SDs of these changes were computed using the RevMan calculator with a correlation value set at 0.5 [24].

For studies that presented results in a graphical format, the WebPlotDigitizer program [25] was used to extract relevant data. If the original publications contained unclear data, attempts were made to contact the corresponding authors via email for clarification. Furthermore, in studies examining multiple dosages of rice bran intervention, the highest dosage was selected for analysis, as it is expected to reflect the maximum potential efficacy of rice bran on the outcomes of interest.

### 2.4. Risk of Bias Assessment

The Cochrane Risk of Bias 2.0 (RoB2) tool for parallel group randomized trials was used to assess the risk of bias across five domains: (1) the randomization process, (2) deviations from intended interventions, (3) missing outcome data, (4) measurement of the outcome, and (5) selection of the reported result. Each study was categorized as having a low risk of bias, some concerns, or a high risk of bias [26].

### 2.5. Quality of Evidence Assessment

To evaluate the quality of the included parameters, the Grading of Recommendations, Assessment, Development, and Evaluation (GRADE) criteria [27] were applied. The parameters were classified into four levels of certainty: high (indicating strong confidence that the estimate closely approximates the true effect), moderate (indicating moderate confidence, though the true effect may differ substantially), low (indicating limited confidence in the estimate’s accuracy), and very low (indicating very low confidence, with a high likelihood that the true effect differs significantly from the estimate). This entire process was conducted independently by two authors.

### 2.6. Data Analysis

Statistical analyses were conducted using RStudio (R version 4.4.0, Boston, MA, USA) with the “meta” package. Numerical data are presented as means with SDs. The pooled weighted mean difference (WMD) with 95% CIs was calculated using a random-effects model with the restricted maximum-likelihood method. Statistical significance was defined as a *p*-value < 0.05. Between-study heterogeneity was evaluated using Cochran’s Q test and the I^2^ statistic. Moderate heterogeneity was defined as an I^2^ value between 30 and 60%, while substantial heterogeneity was indicated by values exceeding 60% [28].

Subgroup analyses were conducted based on participants’ health conditions (dyslipidemia, type 2 diabetes, hypertension, obesity, MetS, and healthy individuals), rice bran dosage (≥20 g/day, <20 g/day, and oryzanol), and the duration of intervention (≥90 days or <90 days). Additionally, for continuous outcomes involving more than six studies, meta-regression analysis was performed to examine the effects of rice bran dosage and intervention duration on each parameter.

Publication bias was evaluated using a funnel plot, and Egger’s test was applied to detect small-study effects. Forest and funnel plots were generated using the “meta” package, while the “ggplot2” package was used to create the heat map.

## 3. Results

### 3.1. Search Results

**Figure 1:**
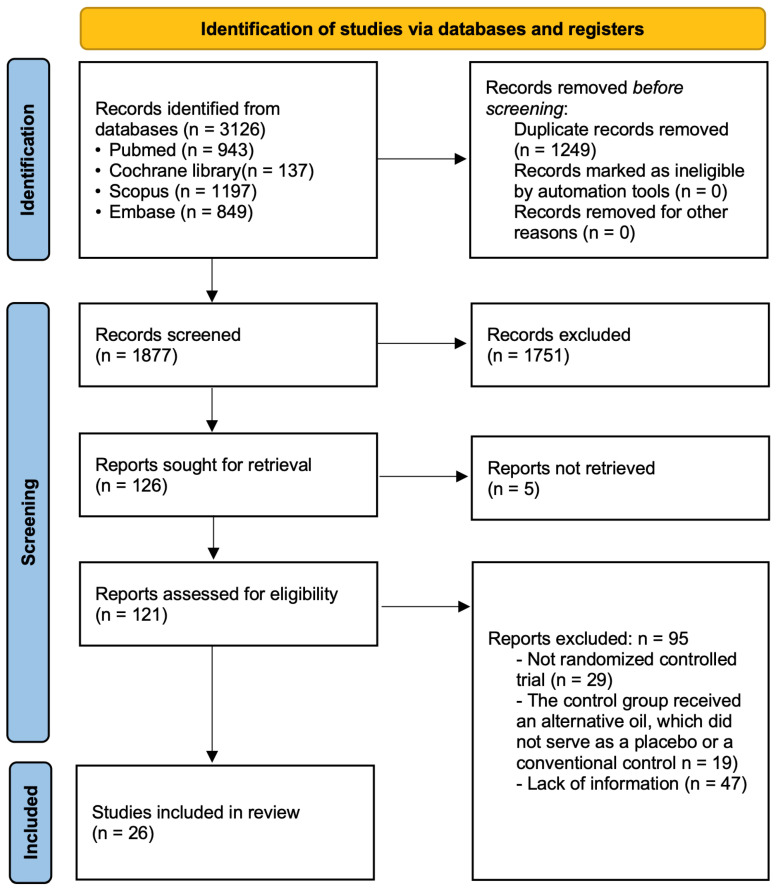
PRISMA flow diagram of the present study.

This study identified 3126 articles through a systematic search using predefined search terms. After duplicate removal, 1877 articles remained. Titles and abstracts were screened, and studies not meet the eligibility criteria were excluded. Subsequently, 121 articles underwent full-text assessment, of which 95 did not fulfill the inclusion criteria. Finally, 26 articles were included in this systematic review. The stages of study selection are summarized in the PRISMA flow diagram, as shown in Figure 1.

### 3.2. Study Characteristics and Risk of Bias Assessment

The study included 26 articles, comprising 642 participants in the rice bran group and 613 in the control group. The majority of studies were conducted in Asia (n = 18), followed by North America (n = 6) and Europe (n = 2). The study populations included healthy individuals (n = 2), individuals with dyslipidemia (n = 11), individuals with type 2 diabetes (n = 4), metabolic syndrome (n = 3), overweight or obesity (n = 1), colorectal cancer (n = 1), hypertension (n = 2), and others, including overweight/obese individuals with hypercholesterolemia (n = 2). The duration of rice bran supplementation ranged from 28 to 180 days, with 84 days being the most commonly studied period. The administered doses of rice bran varied from 1 to 84 g/d, while the doses of its bioactive compounds supplemented in the formulation or diet ranged from 0.04 to 0.3 g/d (Table 1 and Supplementary Material S16). Regarding the risk of bias assessment, seven studies had a high risk of bias, ten had some concerns, and nine had a low risk of bias (Table 1 and Supplementary Material S16).

### 3.3. Effects of Rice Bran on MetS-Related Parameters

#### 3.3.1. Effects of Rice Bran on Anthropometric Parameters

##### Effect of Rice Bran on Body Mass Index Levels

**Figure 2:**
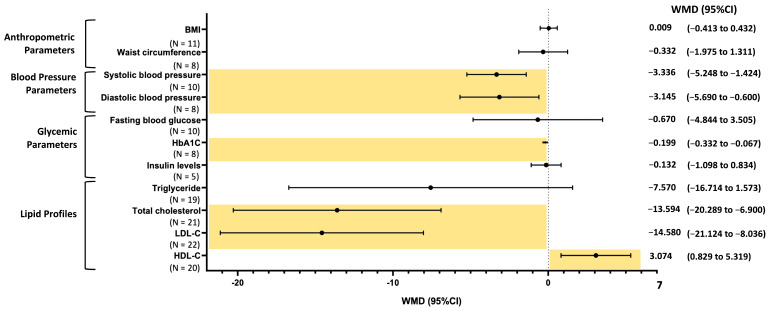
Forest plot summarizing the weighted mean difference (WMD) with 95% confidence intervals (CIs) for metabolic syndrome (MetS)-related parameters following rice bran supplementation compared to the control group.

BMI was analyzed in a total of 11 studies, including 551 participants, with 278 in the intervention group and 273 in the control group. The meta-analysis indicated no significant difference in the rice bran intervention group compared to the control group (WMD: 0.009 kg/m^2^; 95% CI: −0.413 to 0.432; *p* = 0.965) (Figure 2). The results showed substantial heterogeneity (I^2^ = 73%), and no publication bias was detected (Egger’s test: *p* = 0.816) (Supplementary Material S1).

##### Effect of Rice Bran on Waist Circumference

A total of eight clinical trials with a sample size of 217 participants in the intervention groups and 206 in the control groups were included in the meta-analysis. The pooled analysis indicated no significant difference in waist circumference with rice bran consumption compared to the control group (WMD: −0.332 cm; 95% CI: −1.975 to 1.311; *p* = 0.692) (Figure 2), with substantial heterogeneity among studies (I^2^ = 74%). The funnel plot indicated no evidence of publication bias (Egger’s test: *p* = 0.665) (Supplementary Material S2).

#### 3.3.2. Effects of Rice Bran on Blood Pressure Parameters

##### Effect of Rice Bran on Systolic Blood Pressure

A total of 10 studies, comprising 304 participants in the intervention groups and 285 in the control groups, investigated the effects of rice bran on systolic blood pressure (SBP). The pooled analysis demonstrated a significant reduction in SBP with rice bran consumption compared to the control group (WMD: −3.336 mmHg; 95% CI: −5.248 to −1.424; *p* = 0.0006) (Figure 2). Moderate heterogeneity was observed across studies (I^2^ = 39%), and no evidence of publication bias was detected (Egger’s test: *p* = 0.208) (Supplementary Material S3).

##### Effect of Rice Bran on Diastolic Blood Pressure

The effect of rice bran consumption on diastolic blood pressure (DBP) was examined in eight included studies, involving 230 participants in the intervention groups and 212 in the control groups. The pooled results from the random-effects model indicated that rice bran supplementation significantly lowered DBP compared to the control group (WMD: −3.145 mmHg; 95% CI: −5.690 to −0.600; *p* = 0.015) (Figure 2), with substantial heterogeneity across studies (I^2^ = 74%). The funnel plot analysis did not indicate any publication bias (Egger’s test: *p* = 0.053) (Supplementary Material S4).

#### 3.3.3. Effects of Rice Bran on Glycemic Parameters

##### Effect of Rice Bran on Fasting Blood Glucose

The effect of rice bran consumption on fasting blood glucose (FBG) was examined in 10 studies, including 268 participants in the intervention groups and 246 in the control groups. The pooled analysis indicated no significant difference in FBG following rice bran supplementation (WMD: −0.670 mg/dL; 95% CI: −4.844 to 3.505; *p* = 0.753) (Figure 2). The heterogeneity among studies was substantial (I^2^ = 65%), and no evidence of publication bias was detected (Egger’s test: *p* = 0.093) (Supplementary Material S5).

##### Effect of Rice Bran on HbA1c

Eight studies were included to assess the effects of rice bran consumption on HbA1c, with 195 participants in the intervention groups and 188 in the control groups. The pooled analysis demonstrated that rice bran consumption resulted in a significant reduction in HbA1c compared to the control group (WMD: −0.199%; 95% CI: −0.332 to −0.067; *p* = 0.003) (Figure 2), with substantial heterogeneity among studies (I^2^ = 67%). Funnel plot analysis indicated no evidence of publication bias (Egger’s test: *p* = 0.243) (Supplementary Material S6).

##### Effect of Rice Bran on Insulin Levels

Five studies with a total of 207 participants were included in the analysis. The pooled results showed no significant difference in serum insulin levels following rice bran supplementation (WMD: −0.132 μU/mL; 95% CI: −1.098 to 0.834; *p* = 0.788) (Figure 2), with little evidence of heterogeneity among studies (I^2^ = 21%). The funnel plot analysis found no evidence of publication bias in the meta-analysis assessing the effect of rice bran consumption on insulin levels (Egger’s test: *p* = 0.244) (Supplementary Material S7).

#### 3.3.4. Effects of Rice Bran on Lipid Profiles

##### Effect of Rice Bran on Triglyceride Levels

The effect of rice bran on triglyceride levels was evaluated in 19 studies, including 459 participants in the intervention groups and 440 in the control groups. The pooled analysis using a random-effects model revealed no significant difference in triglyceride concentration following rice bran consumption when compared to the control (WMD: −7.570 mg/dL; 95% CI: −16.714 to 1.573; *p* = 0.104) (Figure 2), with considerable heterogeneity observed across studies (I^2^ = 96%). The funnel plot analysis showed no evidence of publication bias in the meta-analysis assessing the effect of rice bran on triglycerides (Egger’s test: *p* = 0.232) (Supplementary Material S8).

##### Effects of Rice Bran on Total Cholesterol Levels

A total of 21 studies from the included studies (517 participants in the intervention groups and 495 in the control groups) examined the effect of rice bran on total cholesterol levels, revealing a significant reduction in serum total cholesterol concentration (WMD: −13.594 mg/dL; 95% CI: −20.289 to −6.900; *p* < 0.0001) (Figure 2), with substantial heterogeneity across studies (I^2^ = 94%). The assessment of publication bias via funnel plot analysis indicated evidence of publication bias in the meta-analysis of rice bran’s effect on total cholesterol (Egger’s test: *p* = 0.011) (Supplementary Material S9).

##### Effect of Rice Bran on LDL-C Levels

In an analysis of 22 studies involving 536 participants who received rice bran and 512 receiving a control, rice bran showed a significant reduction in LDL-C (WMD: −14.580 mg/dL; 95% CI: −21.124 to −8.036; *p* < 0.0001) when compared to the control (Figure 2), with substantial heterogeneity among studies (I^2^ = 97%). Funnel plot analysis revealed evidence of publication bias in the meta-analysis evaluating the effect of rice bran on LDL-C (Egger’s test: *p* = 0.029) (Supplementary Material S10).

##### Effect of Rice Bran on HDL-C Levels

A meta-analysis of 20 studies, involving 487 participants in the intervention groups and 467 in the control groups, indicated that rice bran consumption resulted in a significant increase in HDL-C (WMD: 3.074 mg/dL; 95% CI: 0.829 to 5.319; *p* = 0.007) (Figure 2), with high heterogeneity among studies (I^2^ = 97%). Funnel plot analysis indicated no evidence of publication bias (Egger’s test: *p* = 0.083) (Supplementary Material S11).

### 3.4. Subgroup Analysis

Subgroup analyses were conducted based on participants’ health conditions, rice bran dosage, and the duration of the intervention.

#### 3.4.1. Participants’ Health Conditions

**Figure 3:**
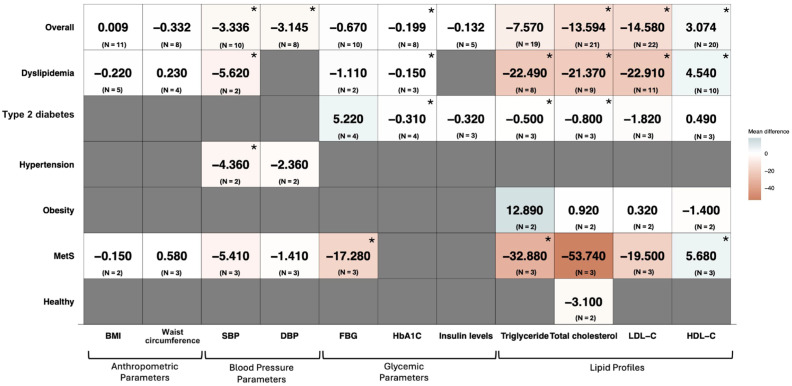
The heatmap illustrates the weighted mean difference (WMD) in metabolic syndrome (MetS)-related parameters between the rice bran intervention and control groups, along with the corresponding sample sizes (n) used in the analysis. The *x*-axis represents MetS-related parameters, while the *y*-axis categorizes subgroups based on participant characteristics. The color gradient in the heatmap indicates the magnitude of the effect. An asterisk (*) marks statistically significant differences at *p* < 0.05.

In Figure 3, a reduction in blood pressure parameters is observed in a subgroup analysis of individuals with dyslipidemia, with significant reductions in SBP following rice bran consumption (WMD: −5.620 mmHg; 95% CI: −10.760 to −0.480) and in individuals with hypertension (WMD: −4.360 mmHg; 95% CI: −7.060 to −1.650) (Supplementary Material S12c).

In terms of glycemic improvement, rice bran supplementation resulted in a significant decrease in FBG in individuals with MetS (WMD: −17.280 mg/dL; 95% CI: −30.900 to −3.660) (Supplementary Material S12e). Reductions in HbA1c levels were also noted, with significant decreases in dyslipidemic participants (WMD: −0.150%; 95% CI: −0.240 to −0.060) and in individuals with type 2 diabetes (WMD: −0.310%; 95% CI: −0.500 to −0.130) (Supplementary Material S12f).

Lipid-lowering effects were observed across various health conditions. Significant reductions in triglycerides were noted in individuals with dyslipidemia (WMD: −22.490 mg/dL; 95% CI: −24.570 to −20.420), type 2 diabetes (WMD: −0.500 mg/dL; 95% CI: −0.630 to −0.370), and MetS (WMD: −32.880 mg/dL; 95% CI: −51.700 to −14.070) (Supplementary Material S12h). Additionally, a significant reduction in total cholesterol was observed in dyslipidemic individuals (WMD: −21.370 mg/dL; 95% CI: −28.360 to −14.380) and in type 2 diabetes patients (WMD: −0.800 mg/dL; 95% CI: −0.930 to −0.670) (Supplementary Material S12i). Moreover, a significant decrease in LDL-C was observed specifically in participants with dyslipidemia (WMD: −22.91 mg/dL; 95% CI: −30.89 to −14.93) (Supplementary Material S12j). Lastly, rice bran supplementation significantly improved HDL-C levels in both the dyslipidemia (WMD: 4.540 mg/dL; 95% CI: 1.420 to 7.660) and MetS groups (WMD: 5.680 mg/dL; 95% CI: 3.430 to 7.940) (Supplementary Material S12k).

#### 3.4.2. Rice Bran Dosage (≥20 g/d, <20 g/d, or Oryzanol)

Subgroup analysis based on rice bran dosage (≥20 g/d, <20 g/d, or oryzanol) revealed that the high dosage of ≥20 g/d significantly impacted various MetS-related parameters. Specifically, a significant reduction in both SBP (WMD: −4.820 mmHg; 95% CI: −8.880 to −0.760) and DBP (WMD: −4.840 mmHg; 95% CI: −7.970 to −1.700) was observed. Additionally, a significant reduction in HbA1c was noted in participants consuming ≥20 g/d of rice bran (WMD: −0.250%; 95% CI: −0.430 to −0.070). This dosage also resulted in significant improvements in lipid profiles, including reductions in total cholesterol (WMD: −12.720 mg/dL; 95% CI: −21.130 to −4.320) and LDL-C (WMD: −12.000 mg/dL; 95% CI: −20.420 to −3.590) (Supplementary Material S15).

For participants receiving supplemented oryzanol (ranging from 0.04 to 0.3 g/d), improvements were observed in several MetS-related parameters, including a reduction in BMI (WMD: −0.490 kg/m^2^; 95% CI: −0.810 to −0.170), SBP (WMD: −4.760 mmHg; 95% CI: −8.250 to −1.270), triglycerides (WMD: −18.920 mg/dL; 95% CI: −29.790 to −8.050), and LDL-C (WMD: −27.770 mg/dL; 95% CI: −50.200 to −5.340). Additionally, oryzanol supplementation led to a significant increase in HDL-C levels (WMD: 8.060 mg/dL; 95% CI: 1.690 to 14.430) (Supplementary Material S15).

#### 3.4.3. Duration of the Intervention (≥90 Days or <90 Days)

Subgroup analysis based on the duration of rice bran supplementation showed that both intervention durations (<90 days and ≥90 days) significantly improved HbA1c, total cholesterol, LDL-C, and HDL-C levels. However, supplementation for ≥90 days further improved BMI (WMD: −0.520 kg/m^2^; 95% CI: −0.900 to −0.140) and triglycerides (WMD: −22.750 mg/dL; 95% CI: −24.860 to −20.640). In contrast, supplementation for <90 days led to additional significant reductions in SBP (WMD: −3.470 mmHg; 95% CI: −5.490 to −1.440) and DBP (WMD: −3.150 mmHg; 95% CI: −5.690 to −0.600) (Supplementary Material S15).

### 3.5. Meta-Regression Analysis

To explore potential associations, a meta-regression analysis was conducted based on the duration of the intervention and rice bran dosage. In terms of intervention duration, the results indicated a significant association between intervention duration and changes in DBP (coefficient = −3.598; 95% CI: −0.283 to −0.084; *p* < 0.001) (Supplementary Material S13d), while no significant associations were observed for other outcomes of interest.

### 3.6. Results of Quality of Evidence Assessment

The quality of evidence varied across outcomes. SBP was rated as moderate-quality, while BMI, WC, FBG, and insulin had a low quality of evidence. DBP, HbA1c, triglycerides, total cholesterol, LDL-C, and HDL-C were classified as very-low-quality evidence (Table 2).

## 4. Discussion

This systematic review and meta-analysis evaluated 26 clinical trials investigating the effects of rice bran supplementation on MetS-related parameters. Our analysis revealed a statistically significant reduction in SBP, DBP, HbA1c, total cholesterol, and LDL-C levels, along with a significant increase in HDL-C levels among rice bran users when compared to the control groups. These findings suggest that rice bran consumption may be beneficial for improving cardiovascular and glycemic markers in individuals with MetS. However, further well-designed long-term clinical trials are warranted to confirm these effects and evaluate their clinical relevance.

This meta-analysis indicates that rice bran has significant blood pressure-lowering effects. The predominant mechanism involves the inhibition of the angiotensin-converting enzyme, which prevents the conversion of angiotensin I into angiotensin II, a potent vasoconstrictor [54]. Furthermore, rice bran has been shown to enhance the production and bioavailability of nitric oxide, promoting vasodilation and further contributing to blood pressure reduction [55,56]. Bioactive compounds in rice bran, such as tocopherols, phenolic acids, and peptides, also play a role in regulating blood pressure through their antioxidant [56] and anti-inflammatory properties [57]. These findings are consistent with previous studies that have demonstrated positive effects on blood pressure regulation, particularly in individuals with either hypertension or obesity combined with hypercholesterolemia [18,50]. Subgroup analysis revealed a significant decrease in blood pressure with interventions lasting less than 90 days, suggesting that rice bran exerts a rapid initial effect on blood pressure. However, the long-term effects (beyond 90 days) were not observed in this study due to a limited number of studies with longer durations. This finding underscores the need for further research to explore the long-term impact of rice bran on blood pressure management.

This meta-analysis also observed a statistically significant reduction in HbA1c levels. A clinically meaningful change in glycemic control is generally defined as a difference of 5 mmol/mol (0.5%) [58]. Although the reduction observed in this study did not reach that clinically significant threshold, it may still indicate potential benefits, as HbA1c reflects long-term glycemic control and provides a reliable assessment of overall glucose metabolism over the preceding two to three months [59]. In contrast, the lack of a significant reduction in FBG may be due to its sensitivity to short-term glycemic fluctuations and its susceptibility to factors such as dietary habits and eating behaviors [60]. Notably, subgroup analysis revealed that individuals with type 2 diabetes, dyslipidemia, or MetS, particularly those with elevated baseline FBG or HbA1c, experienced greater glycemic improvements than healthy participants. In these at-risk groups, higher baseline levels may have made the effects of the intervention more detectable. For instance, Cara et al. found no significant effect in normoglycemic individuals, likely due to their well-regulated glucose homeostasis [61]. These findings suggest that rice bran could be a natural dietary strategy for improving glycemic control in diabetic and at-risk populations [45].

Our findings suggest that rice bran supplementation significantly improves lipid profiles, likely through multiple mechanisms. Rice bran’s bioactive (γ-oryzanol) compounds are known to modulate key metabolic pathways involved in lipid metabolism [16,62]. The reduction in total cholesterol and LDL may be attributed to decreased endogenous cholesterol synthesis, possibly through the downregulation of β-hydroxy-β-methylglutaryl-CoA (HMG-CoA) reductase by γ-Oryzanol [63]. Additionally, rice bran may enhance bile acid excretion, leading to increased fecal cholesterol excretion and reduced intestinal cholesterol absorption [64]. Furthermore, hydrolyzed bound phenolics from rice bran have been shown to inhibit the nuclear receptors involved in lipid metabolism and influence gut microbiota by promoting microbial balance and mitigating dysbiosis, both of which can impact lipid metabolism and HDL levels [65]. Collectively, these mechanisms contribute to the overall improvement in lipid profiles.

The present study observed beneficial effects of rice bran on lipid profiles in the previous analysis, except for triglycerides [21]. Additionally, this meta-analysis revealed no significant effects on waist circumference. These two parameters are closely linked, as triglycerides, a primary form of stored fat, contribute to fat accumulation in the abdominal and visceral areas [21]. The lack of significant changes in triglycerides and waist circumference, despite improvements in other lipid parameters, may be partly attributed to insulin resistance, which impairs the insulin-mediated suppression of hepatic very-low-density lipoprotein (VLDL) production, leading to increased VLDL secretion and altered blood lipid levels [21]. This discrepancy could potentially be caused by variations in the distribution of adipose tissue. While subcutaneous adipose tissue mainly influences waist circumference, visceral adipose tissue is more significantly associated with triglyceride levels and metabolic risk, according to imaging studies employing magnetic resonance imaging or computed tomography. Thus, the unaltered triglycerides and waist circumference may be explained by improvements in other lipid measures without appreciable drops in visceral adipose tissue [21]. The absence of a significant effect could be due to population-specific variations in the regulation of fat storage and metabolism. Specifically, in studies involving participants with type 2 diabetes, only slight reductions in total cholesterol and triglycerides were observed. Type 2 diabetes is characterized by insulin resistance, and elevated oxidative stress levels result in disrupted lipid metabolism compared to the general population [66,67]. As a result, individuals with type 2 diabetes may show smaller responses to interventions such as rice bran supplementation. Similarly, studies on obesity [38,39] have reported different lipid responses. Research in obese adolescents found elevated postprandial triglyceride concentrations and higher triglycerides/apo-B48 ratios, indicating larger triglyceride-rich lipoprotein particles and impaired triglyceride clearance [68]. This suggests that metabolic dysregulation in obesity may limit rice bran’s effectiveness in reducing triglycerides. However, a subgroup analysis revealed that participants with dyslipidemia who had higher baseline lipid levels experienced significant improvements across all lipid parameters. This suggests that individuals with dyslipidemia may respond more favorably to rice bran supplementation, likely due to their more pronounced lipid imbalances at baseline. In the included studies, triglyceride levels were generally assessed from fasting blood samples, as explicitly stated in several trials. However, in one controlled feeding study, blood lipids were collected under standardized conditions on multiple days; although the fasting status was not explicitly reported, lipid measurements in such metabolic feeding trials are typically obtained in the fasting state.

Our analysis also found that higher doses (≥20 g/d) of rice bran or its bioactive compound (i.e., γ-oryzanol) appeared to enhance several MetS-related parameters, including BMI, SBP, DBP, HbA1c, total cholesterol, LDL-C, and HDL-C, suggesting a potential dose-dependent relationship. Consistent with previous meta-analyses, dosages typically ranging from 10 to 30 g/d have been shown to be both safe and effective for health improvements [17,19].

Although this systematic review and meta-analysis provides valuable insights into the effects of rice bran on MetS-related parameters, several limitations should be acknowledged. First, a high degree of heterogeneity was observed in some parameters, which may be attributed to variations in study design, differences in population demographics (such as ethnicity), and inconsistencies in intervention factors, including rice bran form, dosage, duration, and variability in control groups across studies. Second, concerns regarding the quality of evidence should be noted, as approximately half of the evaluated parameters were classified as having very-low-quality evidence. Therefore, these findings should be interpreted with caution. Third, this study was unable to perform a meta-regression analysis on γ-oryzanol concentration due to the limited number of available studies. This limitation restricts the ability to draw strong conclusions about the association between varying oryzanol concentrations and MetS outcomes.

## 5. Conclusions

This systematic review and meta-analysis highlight the potential benefits of rice bran supplementation in improving MetS-related outcomes, including significant reductions in SBP and DBP, HbA1c, total cholesterol, and LDL-C levels, along with an increase in HDL-C levels. Effective doses of rice bran ranged from 1 to 84 g/d, while the doses of its bioactive compounds ranged from 0.04 to 0.3 g/d, suggesting that rice bran has the potential to be a natural dietary component and a promising functional food for managing MetS. However, future research is needed to confirm these findings, with a focus on its long-term safety and the molecular mechanisms underlying its effects.

## Figures and Tables

**Table 1:** Study characteristics and risk of bias assessment.

Author (Year)	Country	Participants	Intervention	Control	Duration (Days)	Risk of Bias Assessment					
						D1	D2	D3	D4	D5	Overall
Accinni (2006) [29]	Italy	Dyslipidemic individuals	Oryzanol (0.040 g/d)	Placebo (rice starch)	120	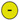	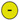	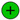	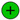	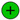	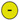
Balters (1981) [30]	United States of America	Healthy individuals	Basal diet with rice bran (20 g/d)	Placebo (Basal diet)	34	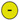	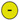	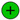	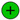	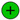	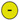
Borresen (2016) [31]	United States of America	Colorectal cancer patients	Rice bran powder (30 g/d)	Placebo (rice starch)	28	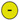	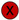	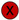	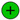	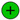	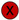
Bumrungpert (2019) [32]	Thailand	Hyperlipidemic individuals	Rice bran oil (30 mL)	Oil without rice bran	28	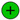	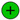	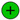	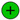	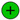	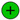
Cheng (2010) [33]	Taiwan	Type 2 diabetes patients	Rice bran oil (3.9 g/d)	Placebo (milled rice flour)	84	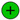	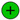	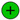	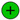	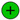	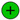
Choi (2014) [34]	Korea	Healthy individuals	Rice bran fermented (3 g/d)	Placebo (rice starch)	56	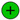	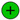	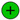	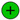	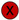	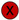
De Lellis (2024) [35]	Italy	Dyslipidemic individuals	ROSSOPURO^®^ Forte with γ-Oryzanol (0.062 g/d)	Placebo	84	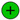	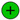	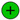	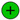	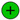	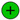
Gerhardt (1998) [36]	United States of America	Hypercholesterolemic individuals	Medium-grain rice bran product (84 g/d)	Placebo (rice starch)	42	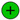	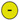	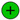	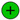	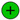	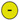
Ghorbani (2025) [37]	Iran	Metabolic syndrome individuals	Standard diet with rice bran powder (15 g/d)	Standard diet without rice bran powder	56	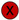	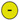	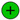	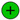	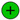	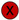
Ito (2015) [38]	Japan	Obese individuals with hypercholesterolemia	Rice bran acylated steryl glucosides (0.05 g/d)	Placebo (rice starch)	84	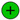	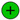	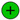	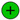	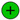	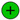
Kim (2008) [39]	Korea	Healthy overweight individuals	Conjugate linoleic acid with oryzanol (0.3 g/d)	Conjugate linoleic acid	84	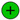	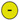	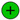	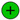	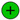	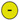
Lai (2012) [40]	Taiwan	Type 2 diabetes patients	Rice bran oil-modified milk (18 g/d)	Placebo (rice starch)	35	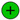	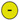	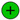	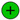	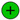	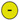
Lin (2020) [19]	Taiwan	Metabolic syndrome and healthy individuals	Refined rice bran (20 g/d)	Refined oil without rice bran	56	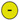	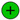	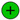	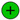	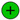	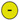
Mahdavi-Roshan (2024) [41]	Iran	Metabolic syndrome individuals	Standard diet with rice bran oil (30 g/d)	Standard diet without rice bran oil	56	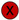	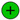	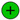	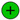	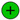	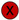
Malve (2010) [42]	India	Hyperlipidemic individuals	Rice bran oil (16.67 g/d)	Blend oil without rice bran oil	90	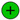	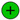	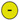	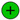	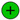	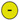
Most (2005) [43]	United States of America	Hypercholesterolemic individuals	Rice bran oil diet (56 g/d)	Control oil blend diet	70	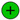	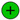	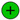	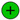	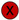	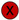
Nhung (2016) [44]	Vietnam	Hypercholesterolemic individuals	Pre-germinated brown rice bran extract (50 g/d)	Placebo (rice starch)	180	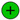	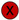	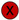	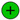	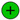	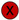
Nikooyeh (2023) [45]	Iran	Type 2 diabetes patients	Oryzanol-fortified canola oil (30 g/d)	Unfortified canola oil (without oryzanol)	84	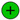	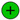	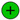	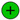	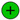	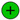
Ogawa (2019) [46]	Japan	High–normal-blood-pressure individuals	Processed rice bran (1 g/d)	Placebo (rice starch)	84	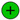	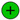	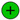	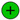	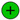	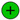
Ogawa (2018) [47]	Japan	High–normal-blood-pressure and mild hypertension individuals	Thermolysin digested rice bran (1 g/d)	Placebo	84	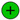	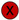	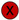	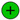	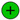	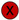
Qureshi (2001) [48]	United States of America	Hypercholesterolemic individuals	Tocotrienol-rich fraction (0.2 g/d)	AHA Step−1 diet	35	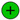	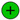	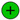	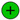	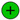	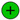
Qureshi (2002) [49]	United States of America	Hypercholesterolemic individuals	Tocotrienol-rich fraction (0.2 g/d)	AHA Step−1 diet	35	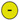	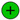	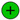	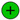	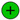	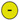
Saphyakhajorn (2022) [50]	Thailand	Overweight/obese individuals with hypercholesterolemia	Defatted rice bran (30 g/d)	Placebo	84	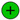	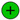	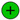	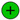	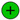	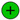
Umin (2015) [51]	Japan	Type 2 diabetes patients	Rice bran oil (8.2 g/d)	Placebo	84	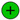	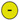	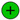	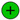	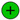	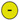
Upadya (2015) [52]	India	Hyperlipidemic individuals	Blend oil with rice bran oil (1 L/person/ month)	Blend oil without rice bran oil	90	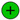	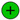	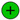	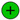	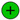	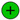
Zavoshy (2012) [53]	Iran	Hyperlipidemic individuals	Low-calorie diet with rice bran oil (30 g/d)	Low-calorie diet without rice bran oil	70	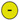	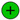	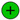	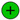	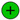	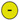

Assessment indicator: 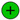 low risk of bias; 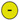 some concerns; 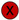 high risk of bias.

**Table 2:** Summary of GRADE’s assessment of evidence quality for the analyzed parameters.

Patient or Population: Participants with Metabolic Syndrome-Related Parameters Intervention: Rice Bran and Its Bioactive Compound Comparison: Control Group											
Study Design	No. of Studies	Certainty Assessment					No. of Participants		Effect		
		Risk of Bias	Inconsistency	Indirectness	Imprecision	Other Considerations	Rice Bran and Its Bioactive Compound	Control	Estimation of Absolute Effects		Certainty
									Relative (95% CI)	Absolute (95% CI)	
Body Mass Index											
RCT	11	Serious ^a^	Not serious	Not serious	Serious ^b^	None	278	273	-	MD 0.009 higher (0.413 lower to 0.432 higher)	⨁⨁◯◯ Low
Waist Circumference											
RCT	8	Serious ^a^	Not serious	Not serious	Serious ^d^	None	217	206	-	MD 0.332 lower (1.975 lower to 1.424 lower)	⨁⨁◯◯ Low
Systolic Blood Pressure											
RCT	10	Serious ^e^	Not serious	Not serious	Not serious	None	304	285	-	MD 3.336 lower (5.248 lower to 1.311 higher)	⨁⨁⨁◯ Moderate
Diastolic Blood Pressure											
RCT	8	Serious ^f^	Very serious ^g^	Not serious	Not serious	None	230	212	-	MD 3.145 lower (5.690 lower to 0.600 lower)	⨁◯◯◯ Very low
Fasting Blood Glucose											
RCT	10	Not serious	Not serious	Not serious	Very serious ^h^	None	268	246	-	MD 0.670 lower (4.844 lower to 3.505 higher)	⨁⨁◯◯ Low
Hemoglobin A1c											
RCT	8	Serious ^a^	Very serious ^i^	Not serious	Not serious	None	195	188	-	MD 0.199 lower (0.332 lower to 0.067 lower)	⨁◯◯◯ Very low
Insulin Level											
RCT	5	Not serious	Not serious	Not serious	Very serious ^j^	None	104	103	-	MD 0.132 lower (1.098 lower to 0.834 higher)	⨁⨁◯◯ Low
Triglycerides											
RCT	19	Serious ^a^	Very serious ^g^	Not serious	Very serious ^k^	None	459	440	-	MD 7.570 lower (16.714 lower to 1.573 higher)	⨁◯◯◯ Very low
Total Cholesterol											
RCT	21	Serious ^a^	Very serious ^g^	Not serious	Not serious	Publication bias strongly suspected ^c^	517	495	-	MD 13.594 lower (20.289 lower to 6.900 lower)	⨁◯◯◯ Very low
Low-Density Lipoprotein											
RCT	22	Serious ^e^	Very serious ^g^	Not serious	Not serious	none	536	512	-	MD 14.580 lower (21.124 lower to 8.036 lower)	⨁◯◯◯ Very low
High-Density Lipoprotein											
RCT	20	Serious ^a^	Very serious ^g^	Not serious	Not serious	none	487	467	-	MD 3.074 lower (0.829 higher to 5.319 higher)	⨁◯◯◯ Very low

CI: confidence interval; MD: mean difference. Explanations: ^a^ Downgraded by one level for risk of bias due to deviations from the intended intervention and missing outcome data. ^b^ Downgraded by two levels for imprecision, as the 95% confidence interval includes both potential benefit and harm (95% CI: −0.413 to 0.432). ^c^ Downgraded by one level for publication bias (*p*-value = 0.011). ^d^ Downgraded by two levels for imprecision, as the 95% confidence interval includes both potential benefit and harm (95% CI: −1.975 to 1.311). ^e^ Downgraded by one level for risk of bias due to deviations from the intended intervention, missing outcome data, and selection of the reported result. ^f^ Downgraded by one level for risk of bias due to selection of the reported result. ^g^ Downgraded by two levels for inconsistency due to considerable heterogeneity. ^h^ Downgraded by two levels for imprecision, as the 95% confidence interval includes both potential benefit and harm (95% CI: −4.844 to 3.505). ^i^ Downgraded by two levels for inconsistency due to substantial heterogeneity. ^j^ Downgraded by two levels for imprecision, as the 95% confidence interval includes both potential benefit and harm (95% CI: −1.098 to 0.834). ^k^ Downgraded by two levels for imprecision, as the 95% confidence interval includes both potential benefit and harm (95% CI: −16.714 to 1.573). Certainty of evidence was rated using GRADE symbols: ⨁⨁⨁◯ (moderate), ⨁⨁◯◯ (low), and ⨁◯◯◯ (very low).

## Data Availability

The datasets used for this study are available from the corresponding author upon reasonable request.

## Supplementary Materials

The following supporting information can be downloaded at: https://www.mdpi.com/article/10.3390/ijms26189051/s1.

## Abbreviations

The following abbreviations are used in this manuscript:

BMI	Body mass index
BP	Blood pressure
CI	Confidence interval
FBG	Fasting blood glucose
GRADE	Grading of Recommendations Assessment, Development, and Evaluation
HbA1c	Hemoglobin A1C
HDL-C	High-density lipoprotein cholesterol
HMG-CoA	β-hydroxy-β-methylglutaryl-CoA
I^2^	I-squared statistic
LDL-C	Low-density lipoprotein cholesterol
MetS	Metabolic syndrome
PRISMA	Preferred Reporting Items for Systematic Reviews and Meta-Analyses
RCTs	Randomized controlled trials
ROB2	Risk of Bias 2 Tool
SBP	Systolic blood pressure
SD	Standard deviation
SE	Standard error
VLDL	Very-low-density lipoprotein
WMD	Weighted mean difference
