# Supplementary material for: Effects of Rice Bran Supplementation on Metabolic Syndrome-Related Parameters: A Systematic Review and Meta-Analysis
Source: Int J Mol Sci. 2025 Sep 17;26(18):9051. doi: 10.3390/ijms26189051 (PMC12469846; doi:10.3390/ijms26189051)
Supplement: Supplementary file 1 [file ijms-26-09051-s001.zip › ijms-3846852-supplementary.pdf]

Supplementary Material S1. Forest plot and funnel plot illustrating the effects of rice bran or its bioactive compound supplementation compared to control groups on body mass index (BMI).

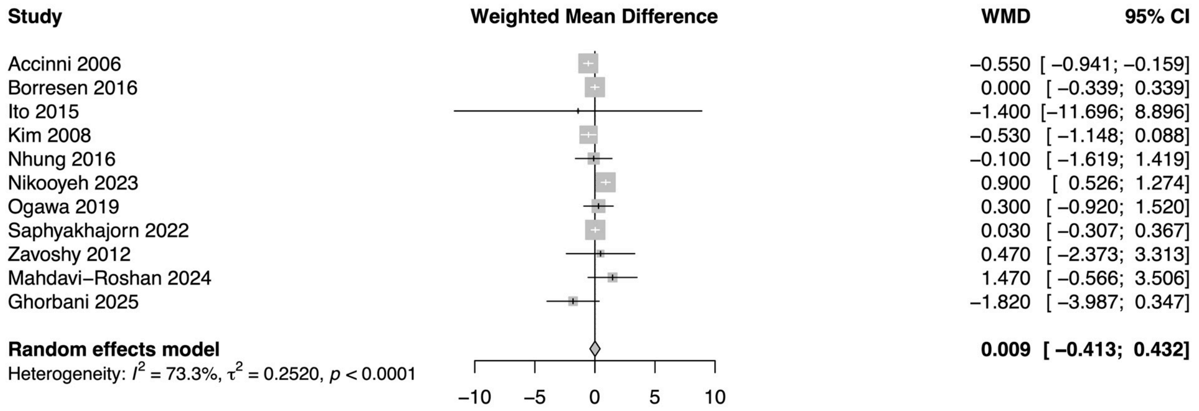

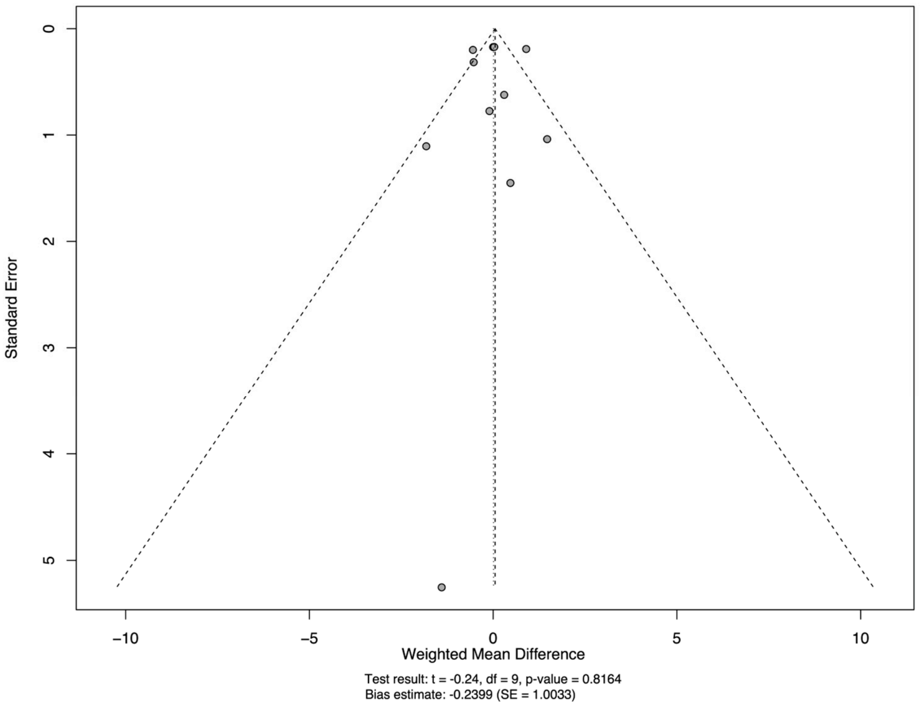

Supplementary Material S2. Forest plot and funnel plot illustrating the effects of rice bran or its bioactive compound supplementation compared to control groups on waist circumference.

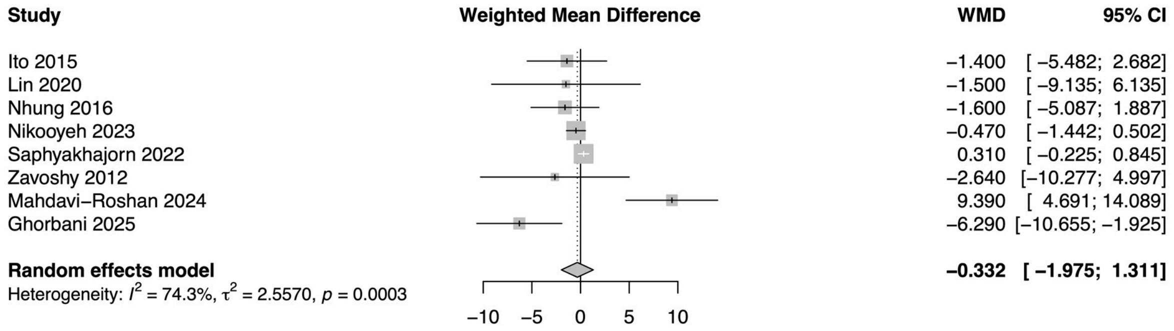

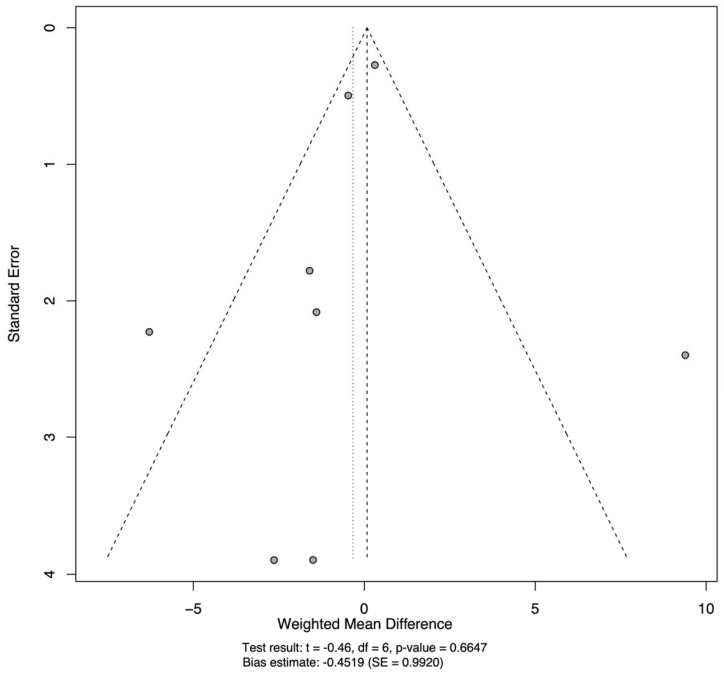

Supplementary Material S3. Forest plot and funnel plot illustrating the effects of rice bran or its bioactive compound supplementation compared to control groups on systolic blood pressure (SBP).

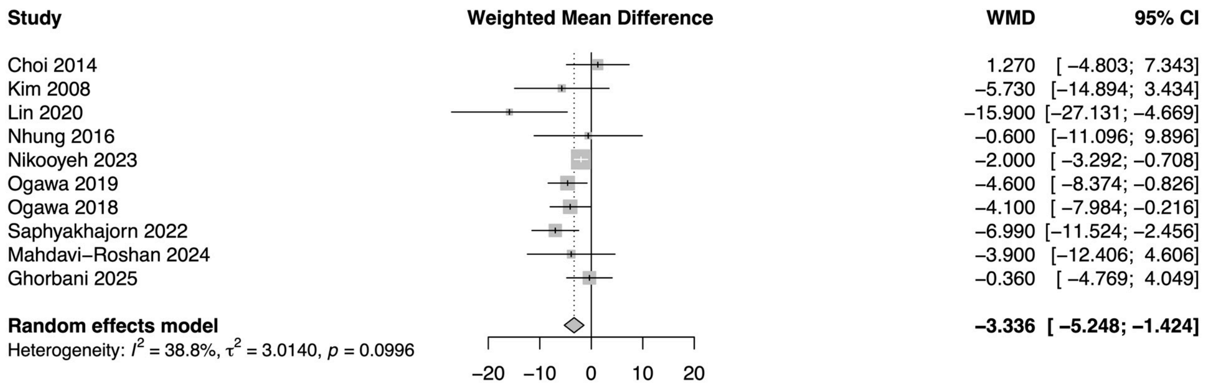

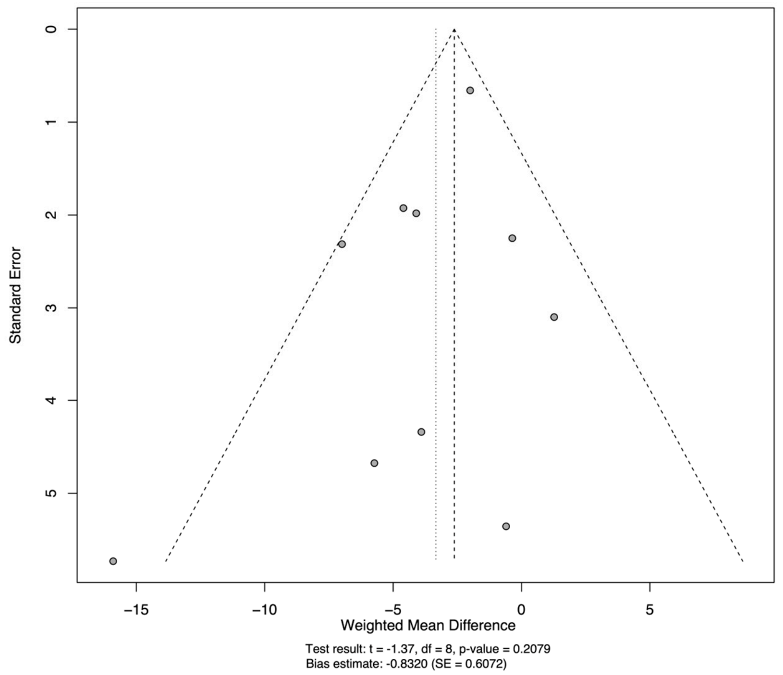

Supplementary Material S4. Forest plot and funnel plot illustrating the effects of rice bran or its bioactive compound supplementation compared to control groups on diastolic blood pressure (DBP).

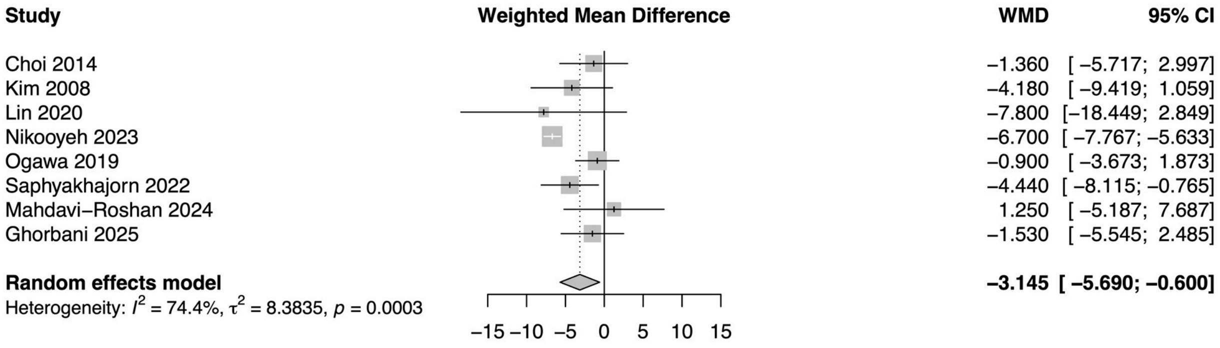

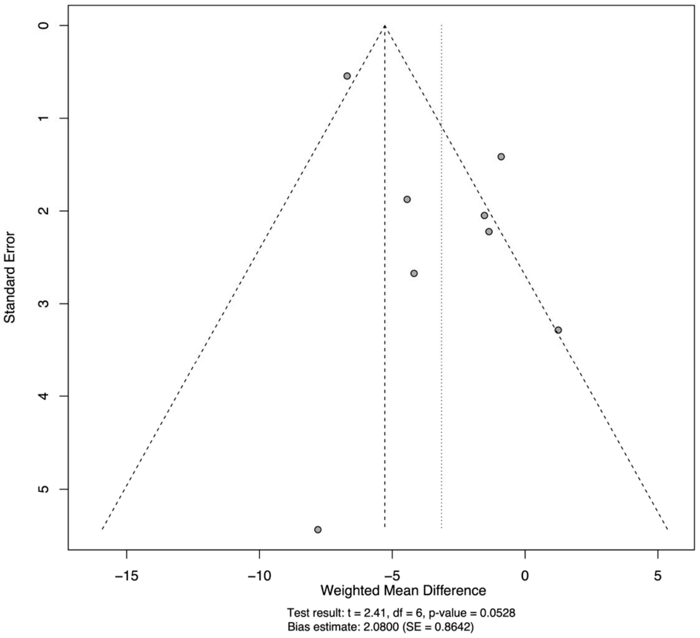

Supplementary Material S5. Forest plot and funnel plot illustrating the effects of rice bran or its bioactive compound supplementation compared to control groups on fasting blood glucose (FBG).

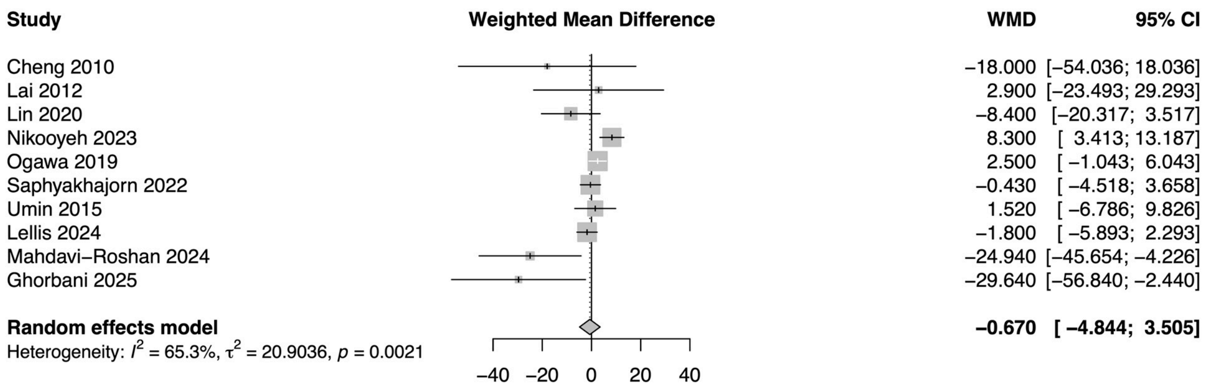

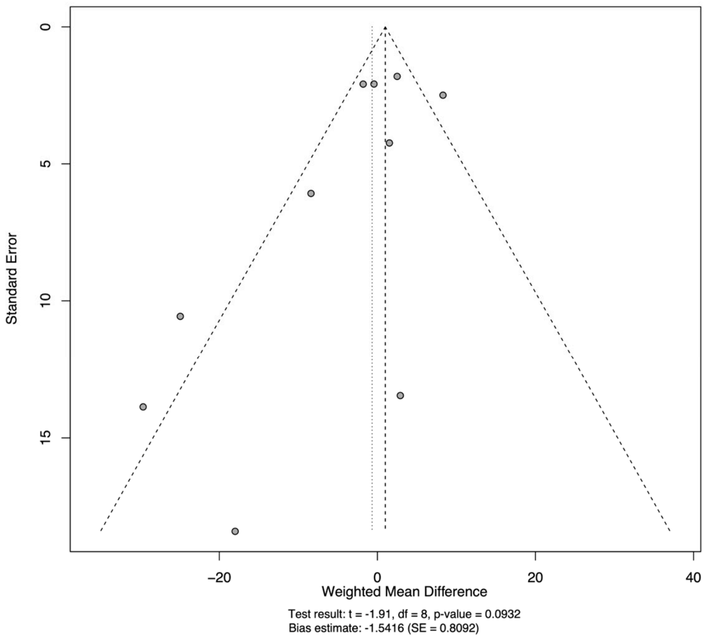

Supplementary Material S6. Forest plot and funnel plot illustrating the effects of rice bran or its bioactive compound supplementation compared to control groups on glycated hemoglobin (HbA1c).

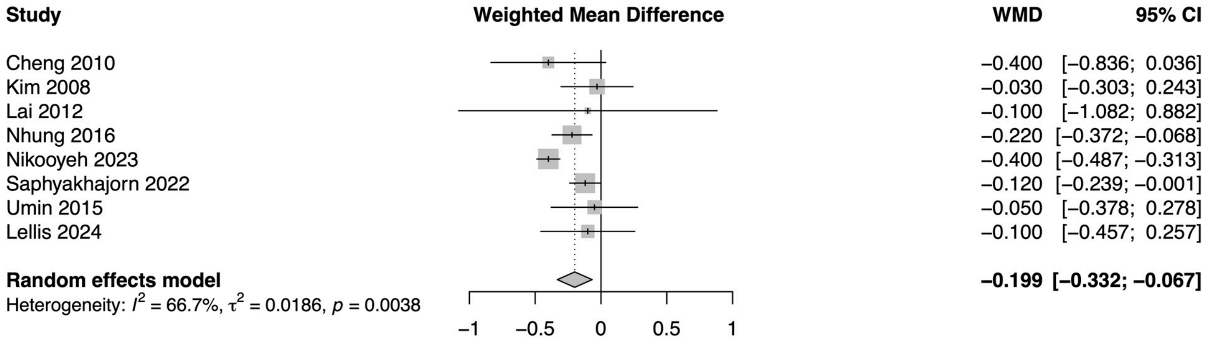

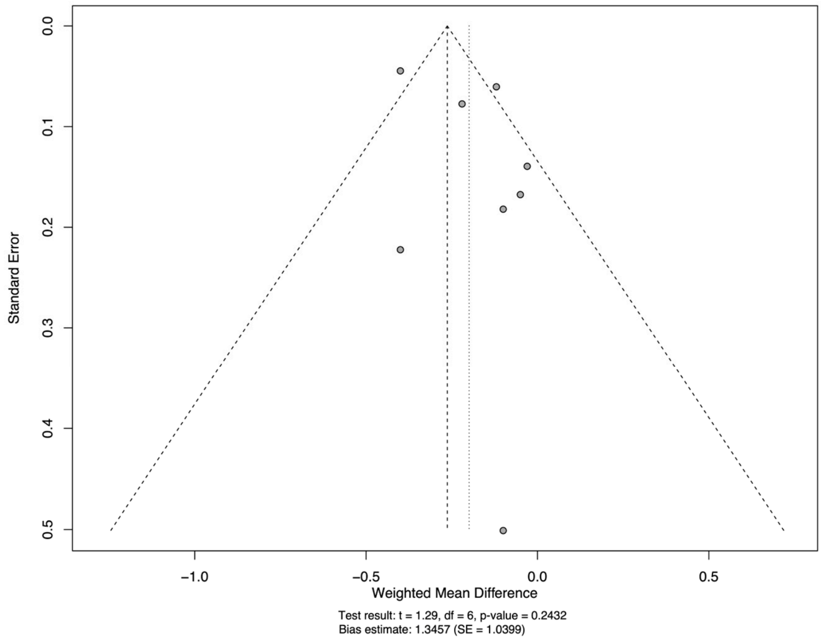

Supplementary Material S7. Forest plot and funnel plot illustrating the effects of rice bran or its bioactive compound supplementation compared to control groups on insulin levels.

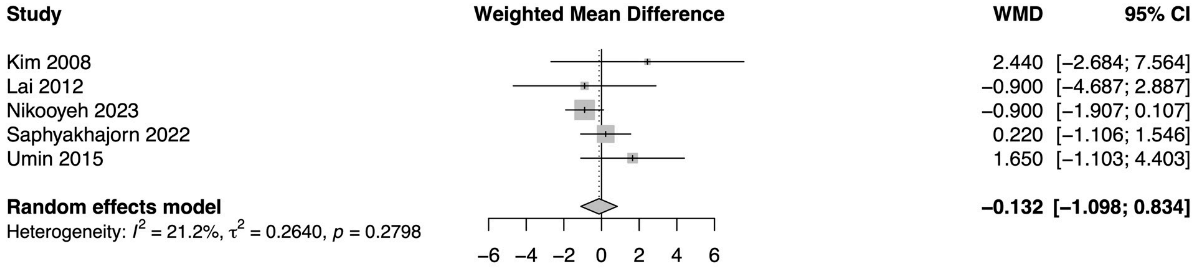

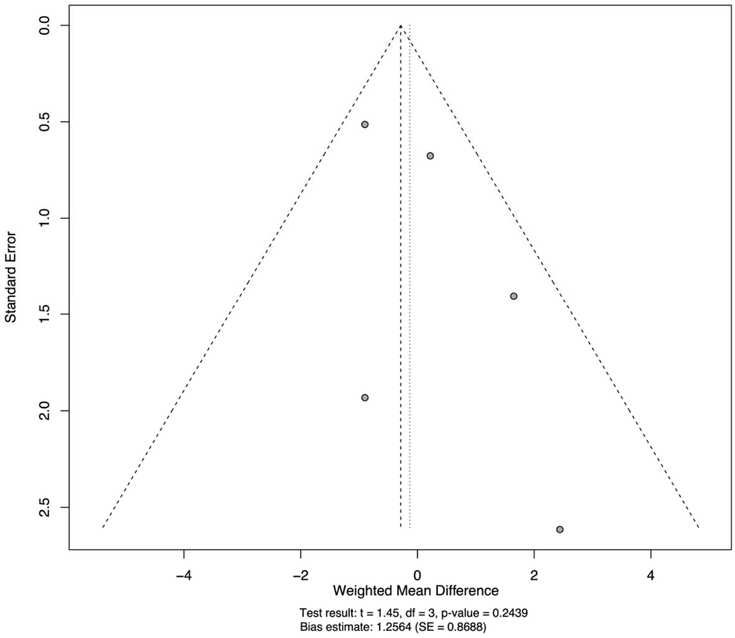

Supplementary Material S8. Forest plot and funnel plot illustrating the effects of rice bran or its bioactive compound supplementation compared to control groups on triglycerides.

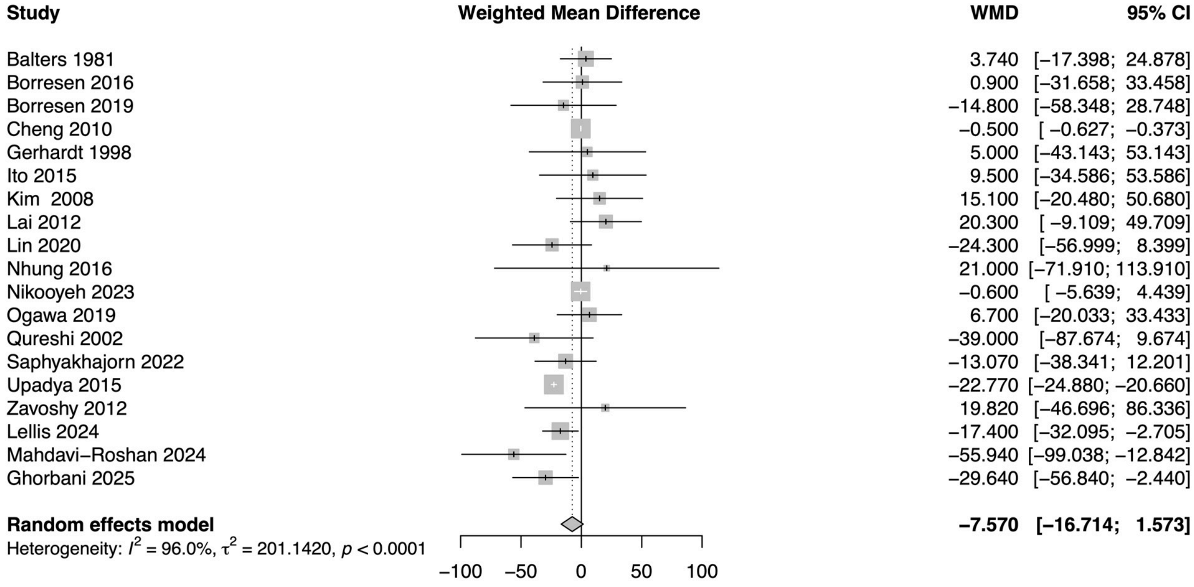

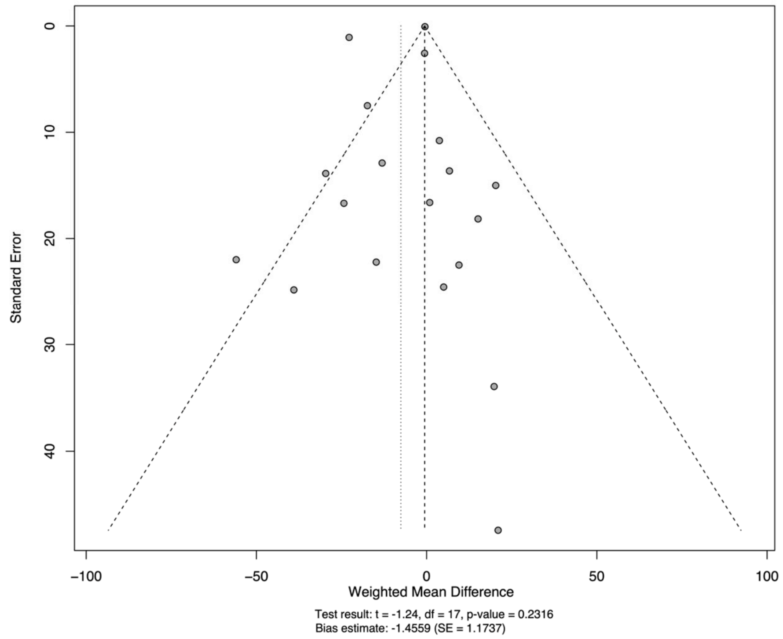

Supplementary Material S9. Forest plot and funnel plot illustrating the effects of rice bran or its bioactive compound supplementation compared to control groups on total cholesterol.

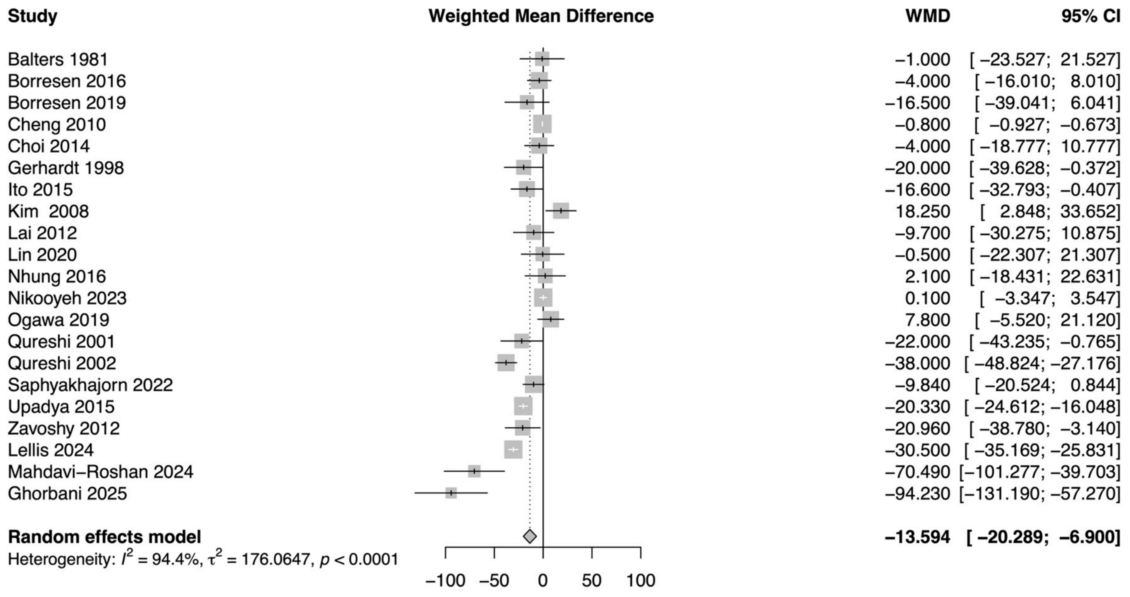

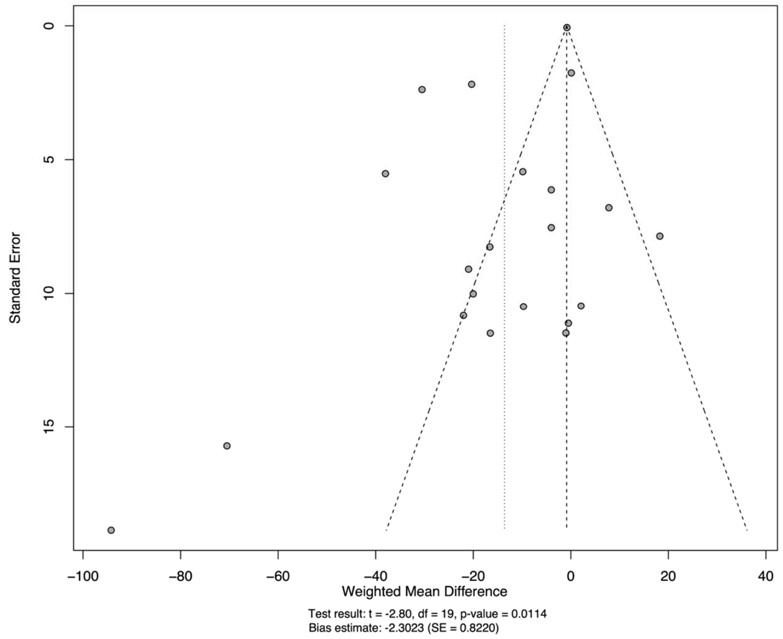

Supplementary Material S10. Forest plot and funnel plot illustrating the effects of rice bran or its bioactive compound supplementation to control groups on low-density lipoprotein cholesterol (LDL-C).

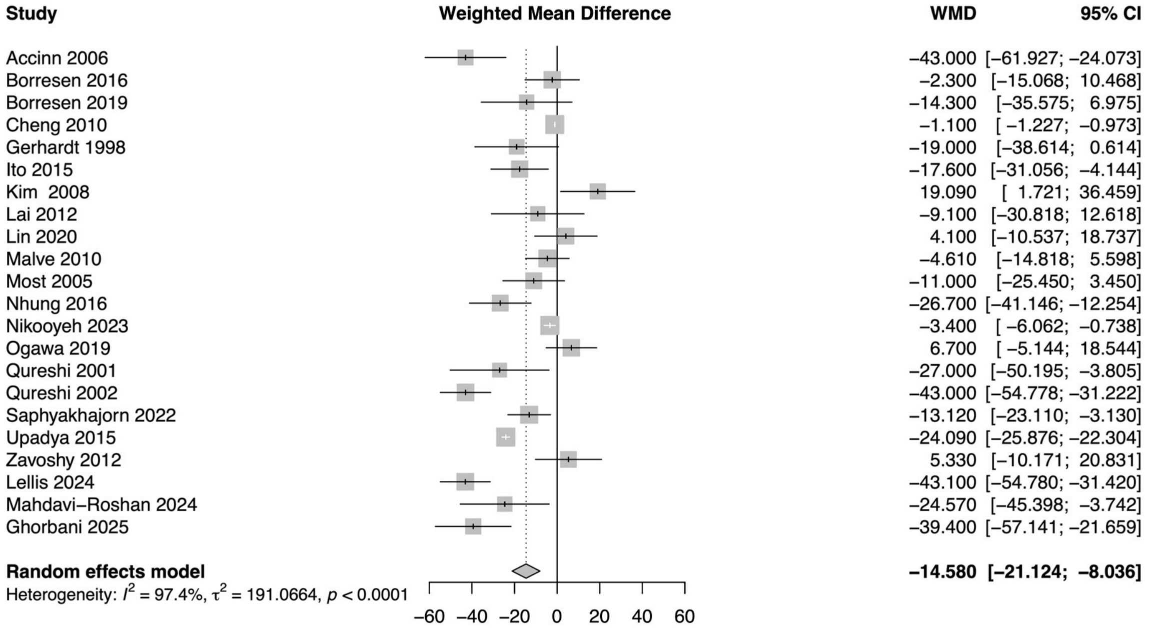

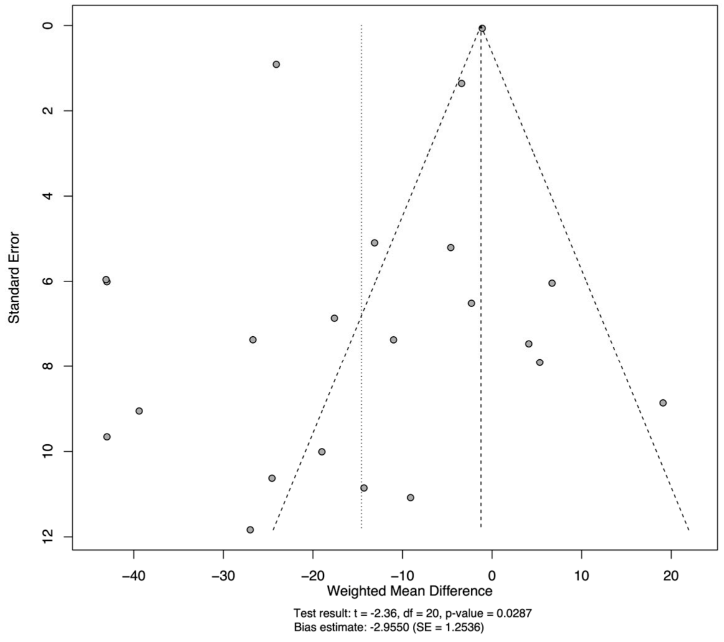

Supplementary Material S11. Forest plot and funnel plot illustrating the effects of rice bran or its bioactive compound supplementation to control groups on high-density lipoprotein cholesterol (HDL-C).

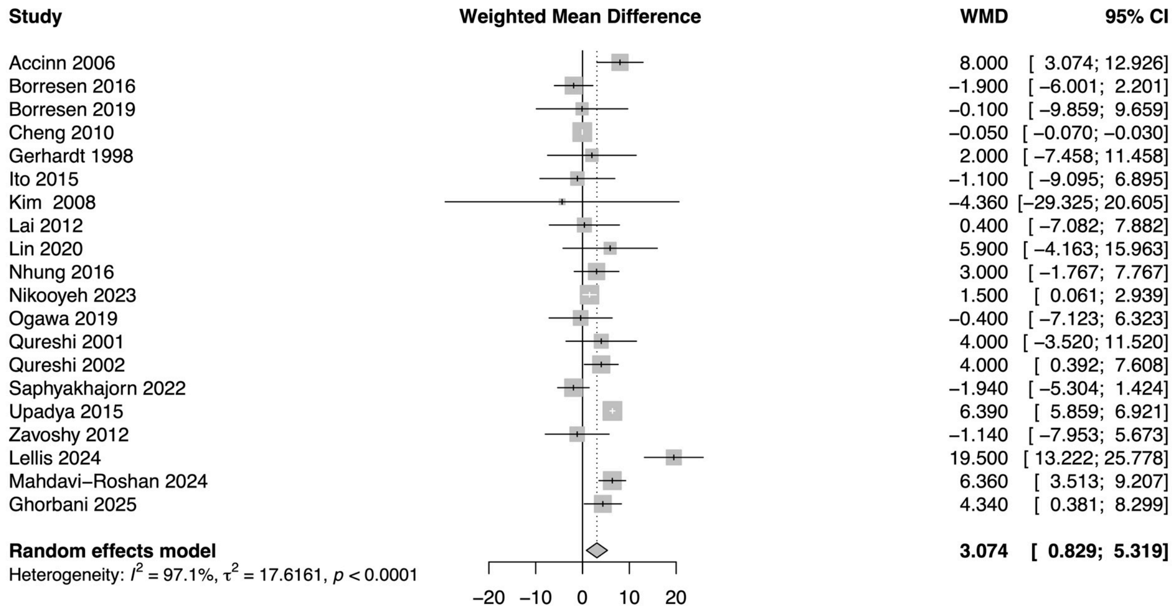

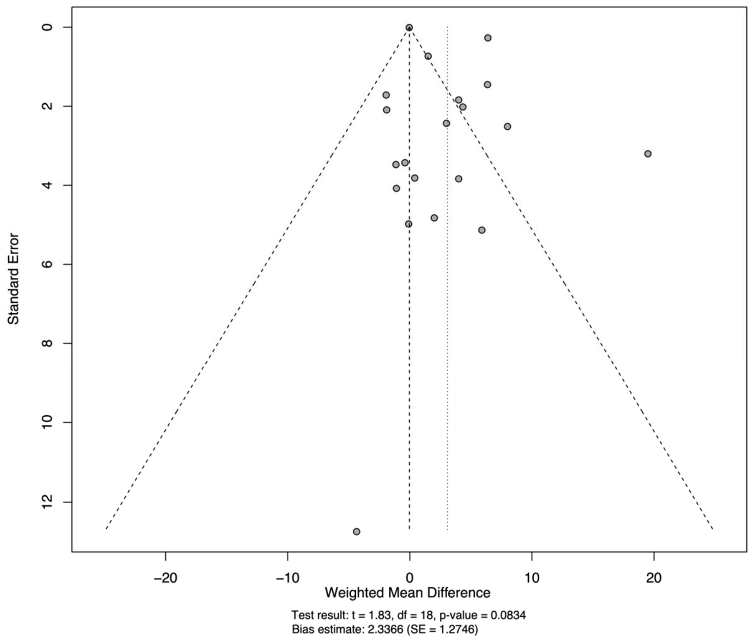

Supplementary Material S12. Subgroup analysis of the effects of rice bran supplementation on various metabolic parameters: (a) body mass index (BMI), (b) waist circumference, (c) systolic blood pressure (SBP), (d) diastolic blood pressure (DBP), (e) fasting blood glucose (FBG), (f) glycated hemoglobin (HbA1c), (g) insulin levels, (h) triglycerides, (i) total cholesterol, (j) low-density lipoprotein cholesterol (LDL-C), and (k) high-density lipoprotein cholesterol (HDL-C).

(a) BMI

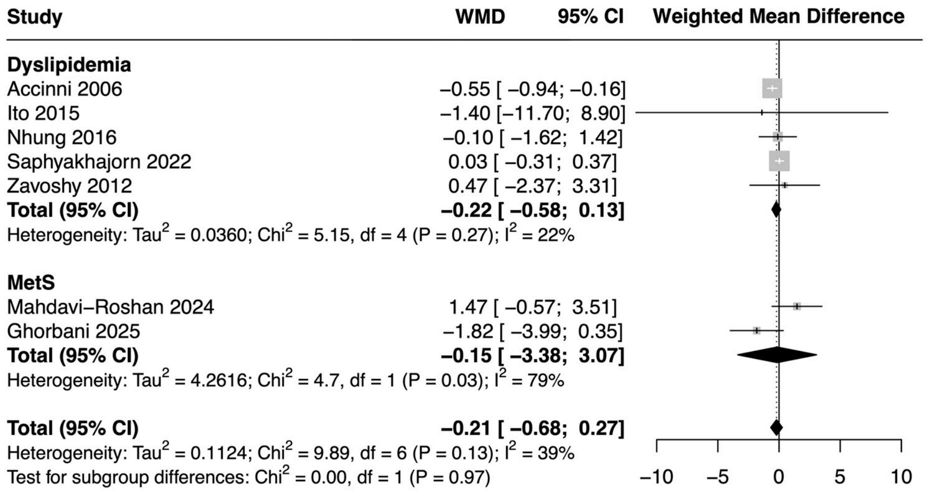

(b) Waist circumference

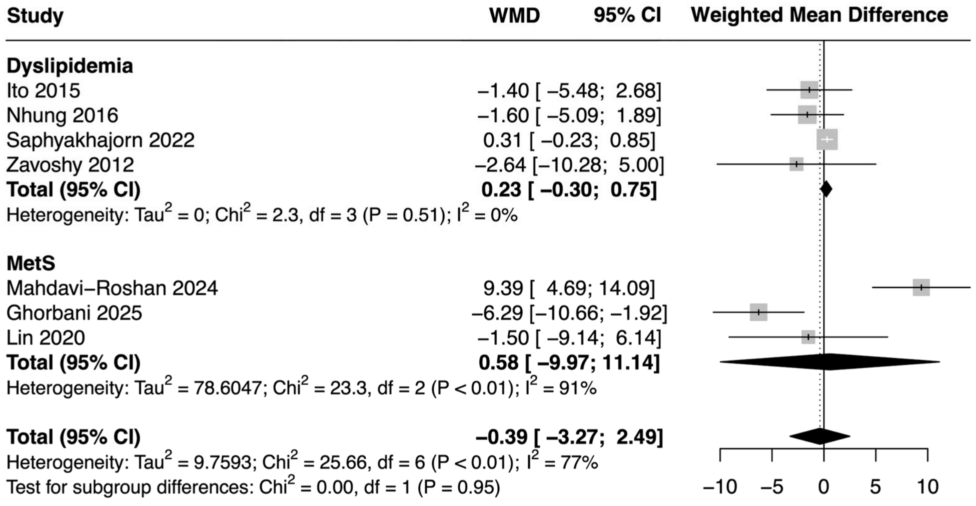

(c) SBP

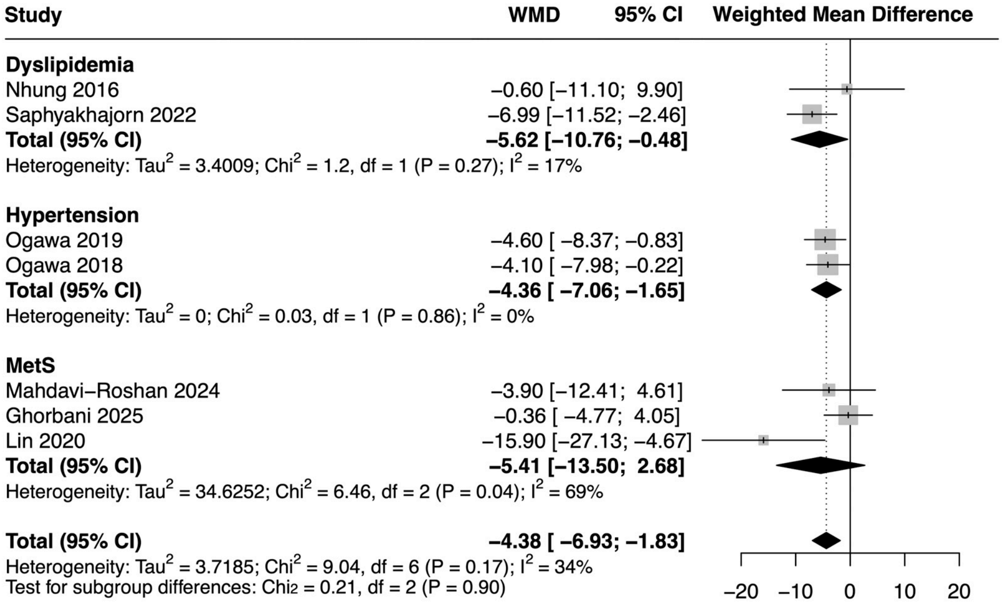
Study
WMD
95% CI
Weighted Mean Difference
Dyslipidemia
Nhung 2016 −0.60 [−11.10; 9.90]
Saphyakhajorn 2022 −6.99 [−11.52; −2.46]
Total (95% CI) −5.62 [−10.76; −0.48]
Heterogeneity: Tau2 = 3.4009; Chi2 = 1.2, df = 1 (P = 0.27); I2 = 17%
Hypertension
Ogawa 2019 −4.60 [−8.37; −0.83]
Ogawa 2018 −4.10 [−7.98; −0.22]
Total (95% CI) −4.36 [−7.06; −1.65]
Heterogeneity: Tau2 = 0; Chi2 = 0.03, df = 1 (P = 0.86); I2 = 0%
MetS
Mahdavi−Roshan 2024 −3.90 [−12.41; 4.61]
Ghorbani 2025 −0.36 [−4.77; 4.05]
Lin 2020 −15.90 [−27.13; −4.67]
Total (95% CI) −5.41 [−13.50; 2.68]
Heterogeneity: Tau2 = 34.6252; Chi2 = 6.46, df = 2 (P = 0.04); I2 = 69%
Total (95% CI) −4.38 [−6.93; −1.83]
Heterogeneity: Tau2 = 3.7185; Chi2 = 9.04, df = 6 (P = 0.17); I2 = 34%
Test for subgroup differences: Chi2 = 0.21, df = 2 (P = 0.90)
−20 −10 0 10 20

(d) DBP

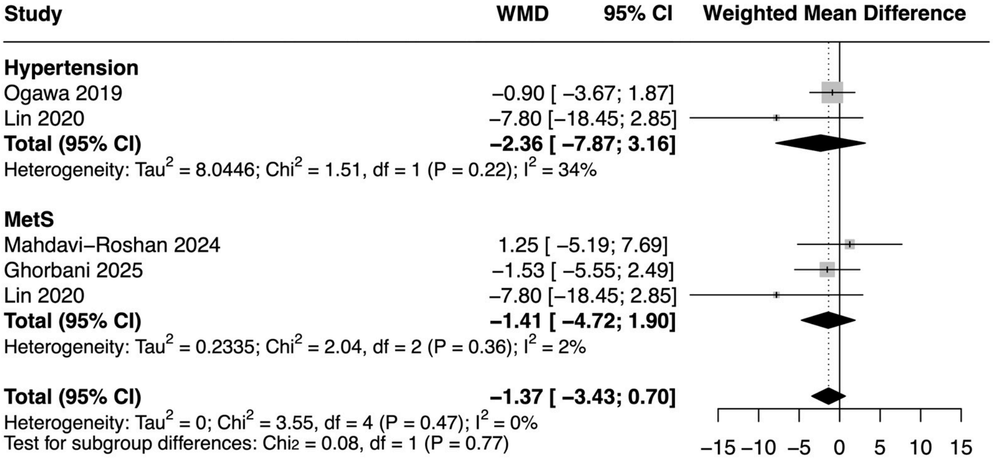
Study
WMD
95% CI
Weighted Mean Difference
Hypertension
Ogawa 2019 −0.90 [−3.67; 1.87]
Lin 2020 −7.80 [−18.45; 2.85]
Total (95% CI) −2.36 [−7.87; 3.16]
Heterogeneity: Tau2 = 8.0446; Chi2 = 1.51, df = 1 (P = 0.22); I2 = 34%
MetS
Mahdavi−Roshan 2024 1.25 [−5.19; 7.69]
Ghorbani 2025 −1.53 [−5.55; 2.49]
Lin 2020 −7.80 [−18.45; 2.85]
Total (95% CI) −1.41 [−4.72; 1.90]
Heterogeneity: Tau2 = 0.2335; Chi2 = 2.04, df = 2 (P = 0.36); I2 = 2%
Total (95% CI) −1.37 [−3.43; 0.70]
Heterogeneity: Tau2 = 0; Chi2 = 3.55, df = 4 (P = 0.47); I2 = 0%
Test for subgroup differences: Chi2 = 0.08, df = 1 (P = 0.77)
−15 −10 −5 0 5 10 15

(e) FBG

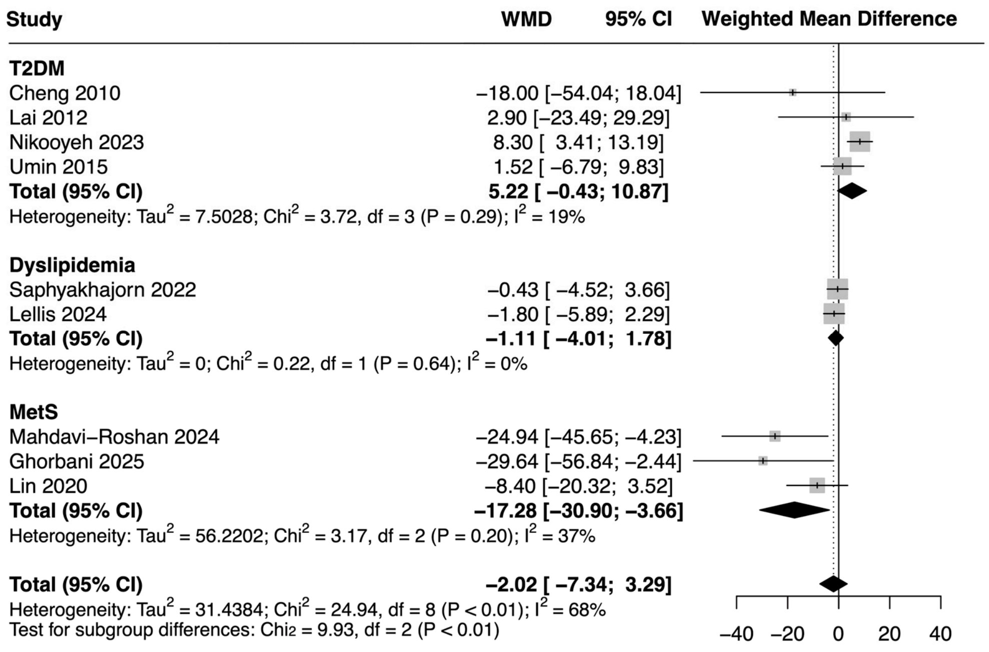

Study
WMD
95% CI
Weighted Mean Difference
T2DM
Cheng 2010 −18.00 [−54.04; 18.04]
Lai 2012 2.90 [−23.49; 29.29]
Nikooyeh 2023 8.30 [3.41; 13.19]
Umin 2015 1.52 [−6.79; 9.83]
Total (95% CI) 5.22 [−0.43; 10.87]
Heterogeneity: Tau^2 = 7.5028; Chi^2 = 3.72, df = 3 (P = 0.29); I^2 = 19%
Dyslipidemia
Saphyakhajorn 2022 −0.43 [−4.52; 3.66]
Lellis 2024 −1.80 [−5.89; 2.29]
Total (95% CI) −1.11 [−4.01; 1.78]
Heterogeneity: Tau^2 = 0; Chi^2 = 0.22, df = 1 (P = 0.64); I^2 = 0%
MetS
Mahdavi−Roshan 2024 −24.94 [−45.65; −4.23]
Ghorbani 2025 −29.64 [−56.84; −2.44]
Lin 2020 −8.40 [−20.32; 3.52]
Total (95% CI) −17.28 [−30.90; −3.66]
Heterogeneity: Tau^2 = 56.2202; Chi^2 = 3.17, df = 2 (P = 0.20); I^2 = 37%
Total (95% CI) −2.02 [−7.34; 3.29]
Heterogeneity: Tau^2 = 31.4384; Chi^2 = 24.94, df = 8 (P < 0.01); I^2 = 68%
Test for subgroup differences: Chi2 = 9.93, df = 2 (P < 0.01)
−40 −20 0 20 40

(f) HbA1C

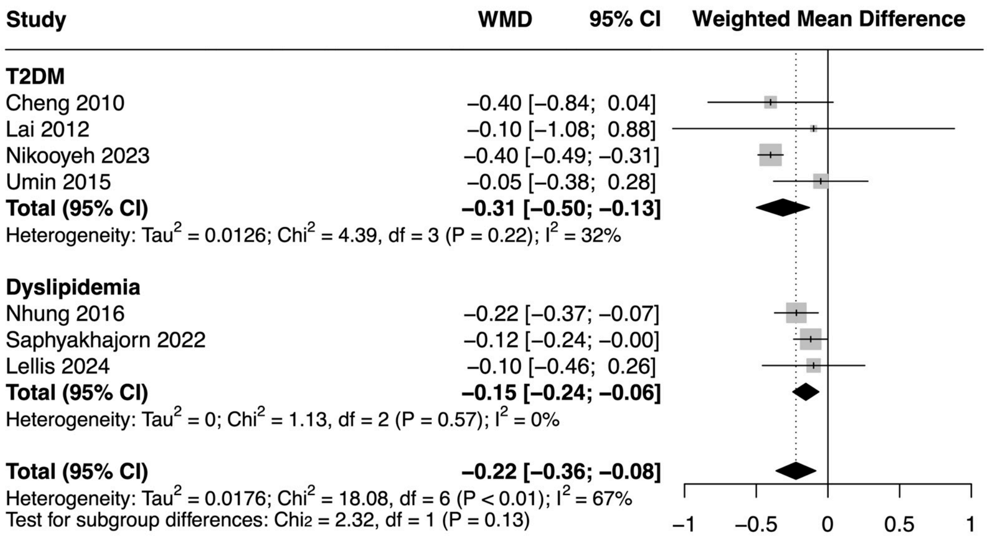

Study
WMD
95% CI
Weighted Mean Difference
T2DM
Cheng 2010 −0.40 [−0.84; 0.04]
Lai 2012 −0.10 [−1.08; 0.88]
Nikooyeh 2023 −0.40 [−0.49; −0.31]
Umin 2015 −0.05 [−0.38; 0.28]
Total (95% CI) −0.31 [−0.50; −0.13]
Heterogeneity: Tau^2 = 0.0126; Chi^2 = 4.39, df = 3 (P = 0.22); I^2 = 32%
Dyslipidemia
Nhung 2016 −0.22 [−0.37; −0.07]
Saphyakhajorn 2022 −0.12 [−0.24; −0.00]
Lellis 2024 −0.10 [−0.46; 0.26]
Total (95% CI) −0.15 [−0.24; −0.06]
Heterogeneity: Tau^2 = 0; Chi^2 = 1.13, df = 2 (P = 0.57); I^2 = 0%
Total (95% CI) −0.22 [−0.36; −0.08]
Heterogeneity: Tau^2 = 0.0176; Chi^2 = 18.08, df = 6 (P < 0.01); I^2 = 67%
Test for subgroup differences: Chi2 = 2.32, df = 1 (P = 0.13)
−1 −0.5 0 0.5 1

(g) Insulin levels

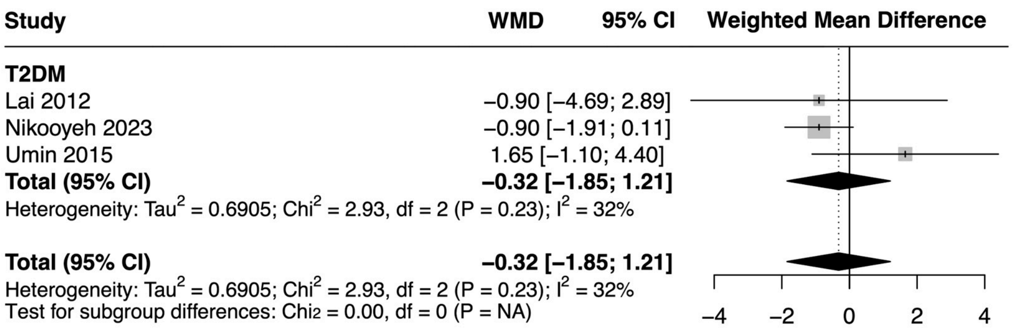

(h) Triglycerides

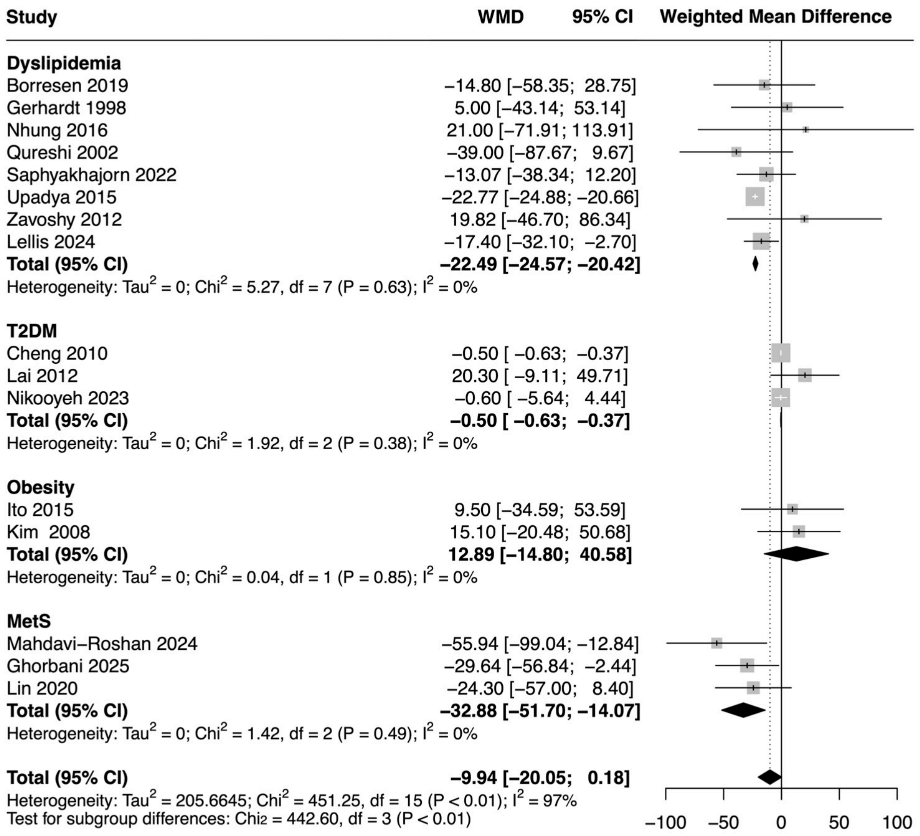

(i) Total cholesterol

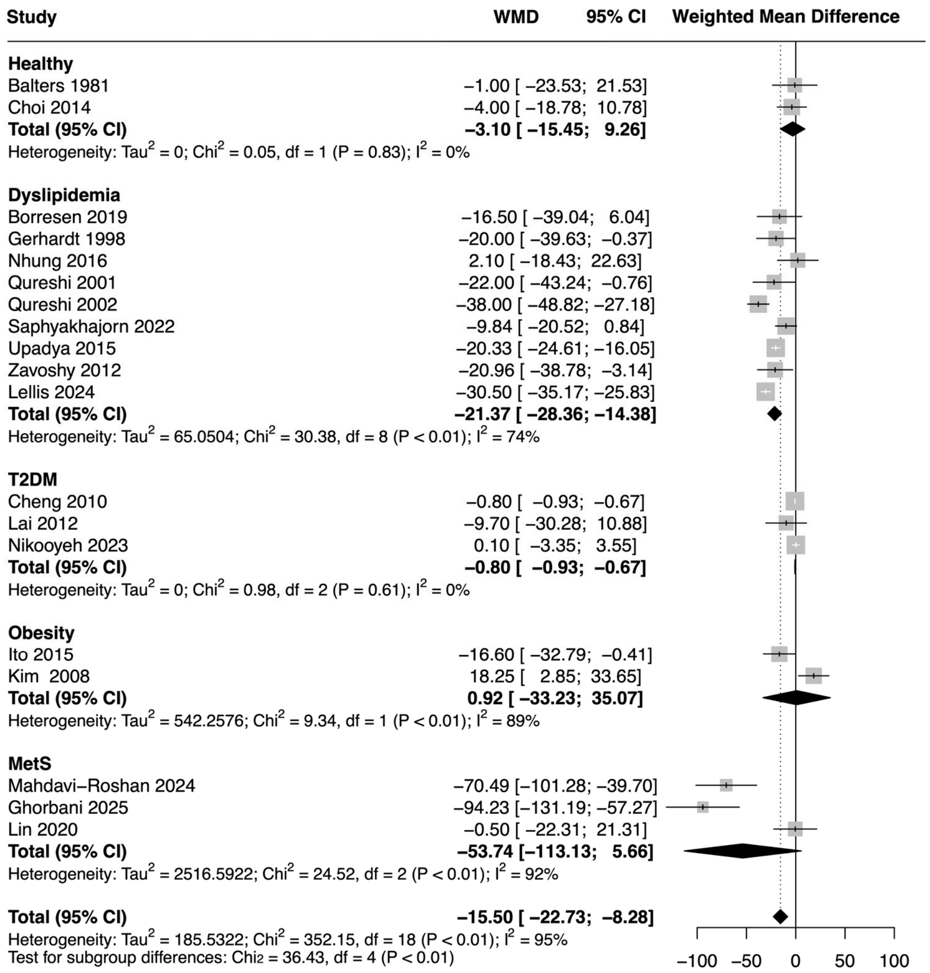

(j) LDL-C

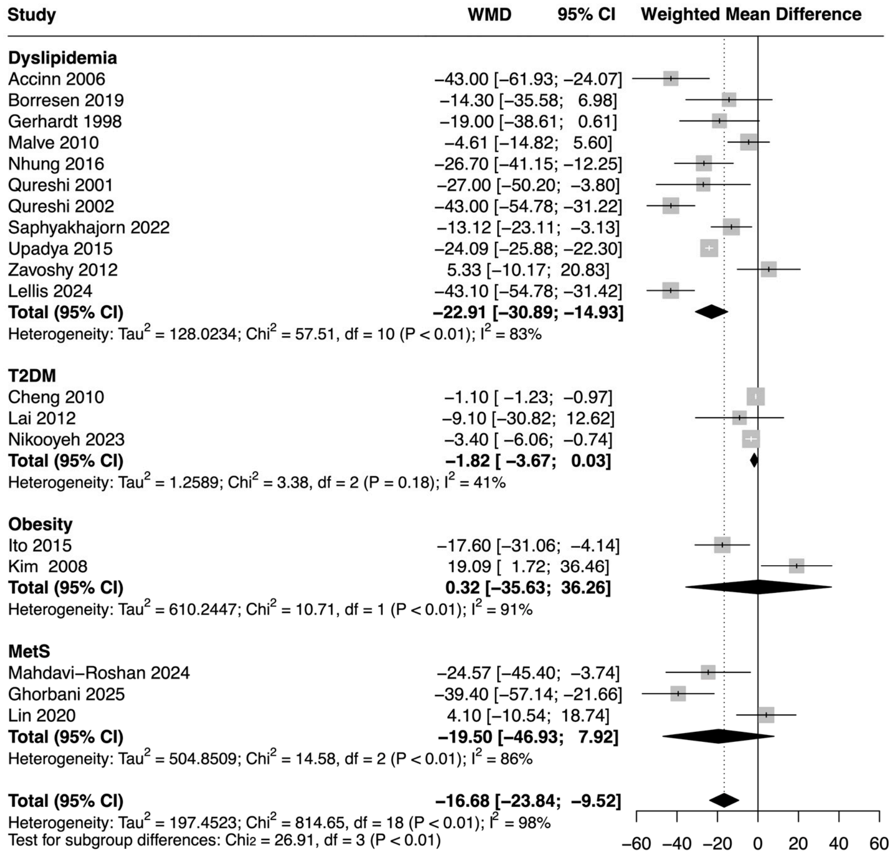
Study
WMD
95% CI
Weighted Mean Difference
Dyslipidemia
Accinn 2006 −43.00 [−61.93; −24.07]
Borresen 2019 −14.30 [−35.58; 6.98]
Gerhardt 1998 −19.00 [−38.61; 0.61]
Malve 2010 −4.61 [−14.82; 5.60]
Nhung 2016 −26.70 [−41.15; −12.25]
Qureshi 2001 −27.00 [−50.20; −3.80]
Qureshi 2002 −43.00 [−54.78; −31.22]
Saphyakhajorn 2022 −13.12 [−23.11; −3.13]
Upadya 2015 −24.09 [−25.88; −22.30]
Zavoshy 2012 5.33 [−10.17; 20.83]
Lellis 2024 −43.10 [−54.78; −31.42]
Total (95% CI) −22.91 [−30.89; −14.93]
Heterogeneity: Tau^2 = 128.0234; Chi^2 = 57.51, df = 10 (P < 0.01); I^2 = 83%
T2DM
Cheng 2010 −1.10 [−1.23; −0.97]
Lai 2012 −9.10 [−30.82; 12.62]
Nikooyeh 2023 −3.40 [−6.06; −0.74]
Total (95% CI) −1.82 [−3.67; 0.03]
Heterogeneity: Tau^2 = 1.2589; Chi^2 = 3.38, df = 2 (P = 0.18); I^2 = 41%
Obesity
Ito 2015 −17.60 [−31.06; −4.14]
Kim 2008 19.09 [1.72; 36.46]
Total (95% CI) 0.32 [−35.63; 36.26]
Heterogeneity: Tau^2 = 610.2447; Chi^2 = 10.71, df = 1 (P < 0.01); I^2 = 91%
MetS
Mahdavi−Roshan 2024 −24.57 [−45.40; −3.74]
Ghorbani 2025 −39.40 [−57.14; −21.66]
Lin 2020 4.10 [−10.54; 18.74]
Total (95% CI) −19.50 [−46.93; 7.92]
Heterogeneity: Tau^2 = 504.8509; Chi^2 = 14.58, df = 2 (P < 0.01); I^2 = 86%
Total (95% CI) −16.68 [−23.84; −9.52]
Heterogeneity: Tau^2 = 197.4523; Chi^2 = 814.65, df = 18 (P < 0.01); I^2 = 98%
Test for subgroup differences: Chi2 = 26.91, df = 3 (P < 0.01)
−60 −40 −20 0 20 40 60

(k) HDL-C

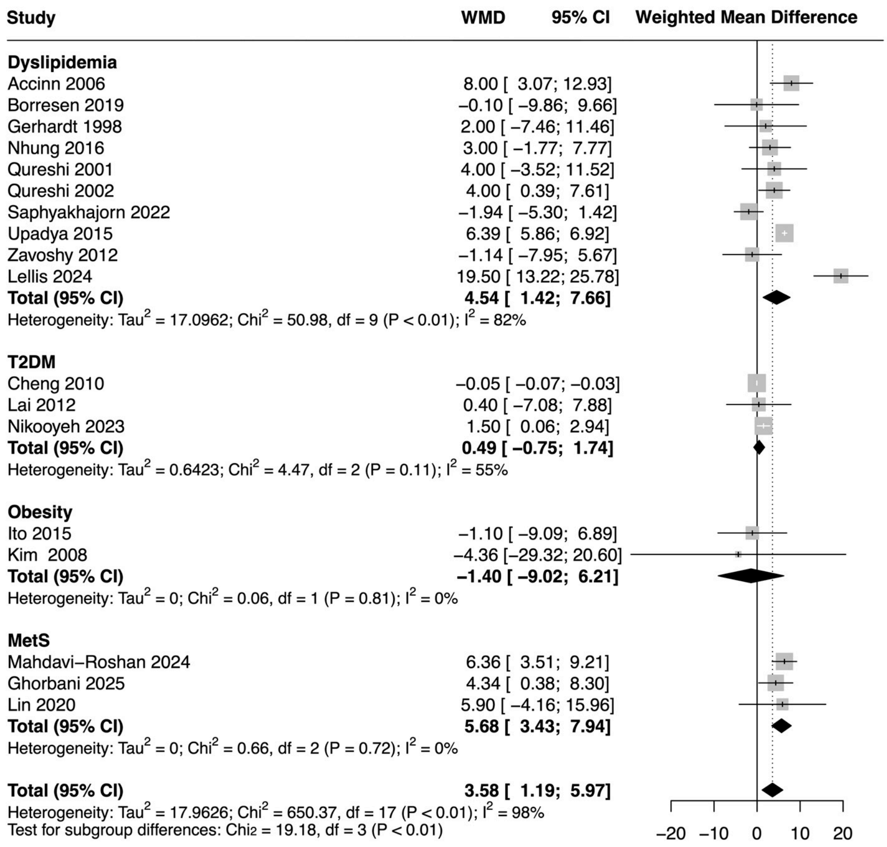
Study
WMD
95% CI
Weighted Mean Difference
Dyslipidemia
Accinn 2006 8.00 [3.07; 12.93]
Borresen 2019 −0.10 [−9.86; 9.66]
Gerhardt 1998 2.00 [−7.46; 11.46]
Nhung 2016 3.00 [−1.77; 7.77]
Qureshi 2001 4.00 [−3.52; 11.52]
Qureshi 2002 4.00 [0.39; 7.61]
Saphyakhajorn 2022 −1.94 [−5.30; 1.42]
Upadya 2015 6.39 [5.86; 6.92]
Zavoshy 2012 −1.14 [−7.95; 5.67]
Lellis 2024 19.50 [13.22; 25.78]
Total (95% CI) 4.54 [1.42; 7.66]
Heterogeneity: Tau2 = 17.0962; Chi2 = 50.98, df = 9 (P < 0.01); I2 = 82%
T2DM
Cheng 2010 −0.05 [−0.07; −0.03]
Lai 2012 0.40 [−7.08; 7.88]
Nikooyeh 2023 1.50 [0.06; 2.94]
Total (95% CI) 0.49 [−0.75; 1.74]
Heterogeneity: Tau2 = 0.6423; Chi2 = 4.47, df = 2 (P = 0.11); I2 = 55%
Obesity
Ito 2015 −1.10 [−9.09; 6.89]
Kim 2008 −4.36 [−29.32; 20.60]
Total (95% CI) −1.40 [−9.02; 6.21]
Heterogeneity: Tau2 = 0; Chi2 = 0.06, df = 1 (P = 0.81); I2 = 0%
MetS
Mahdavi−Roshan 2024 6.36 [3.51; 9.21]
Ghorbani 2025 4.34 [0.38; 8.30]
Lin 2020 5.90 [−4.16; 15.96]
Total (95% CI) 5.68 [3.43; 7.94]
Heterogeneity: Tau2 = 0; Chi2 = 0.66, df = 2 (P = 0.72); I2 = 0%
Total (95% CI) 3.58 [1.19; 5.97]
Heterogeneity: Tau2 = 17.9626; Chi2 = 650.37, df = 17 (P < 0.01); I2 = 98%
Test for subgroup differences: Chi2 = 19.18, df = 3 (P < 0.01)
−20 −10 0 10 20

Supplementary Material S13. Meta-regression analysis of the effect of rice bran supplementation duration (days) on various metabolic parameters: (a) body mass index (BMI), (b) waist circumference, (c) systolic blood pressure (SBP), (d) diastolic blood pressure (DBP), (e) fasting blood glucose (FBG), (f) HbA1C, (g) insulin levels, (h) triglycerides, (i) total cholesterol, (j) low-density lipoprotein cholesterol (LDL-C), and (k) high-density lipoprotein cholesterol (HDL-C). The X-axis represents the duration of rice bran supplementation, while the Y-axis represents the weighted mean difference (WMD) in each metabolic parameter. Each dot represents an individual study, with the regression line indicating the overall trend and the shaded area representing the 95% confidence interval (CI).

(a) BMI

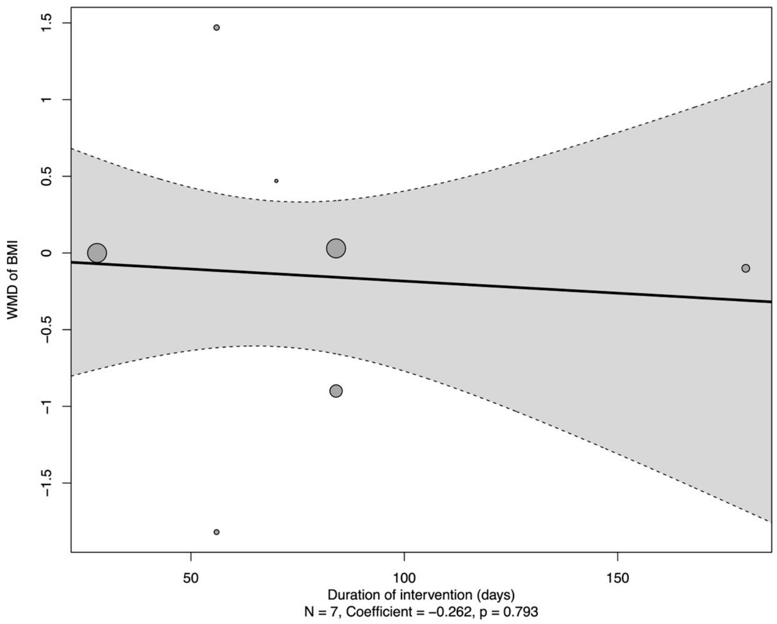

(b) Waist circumference

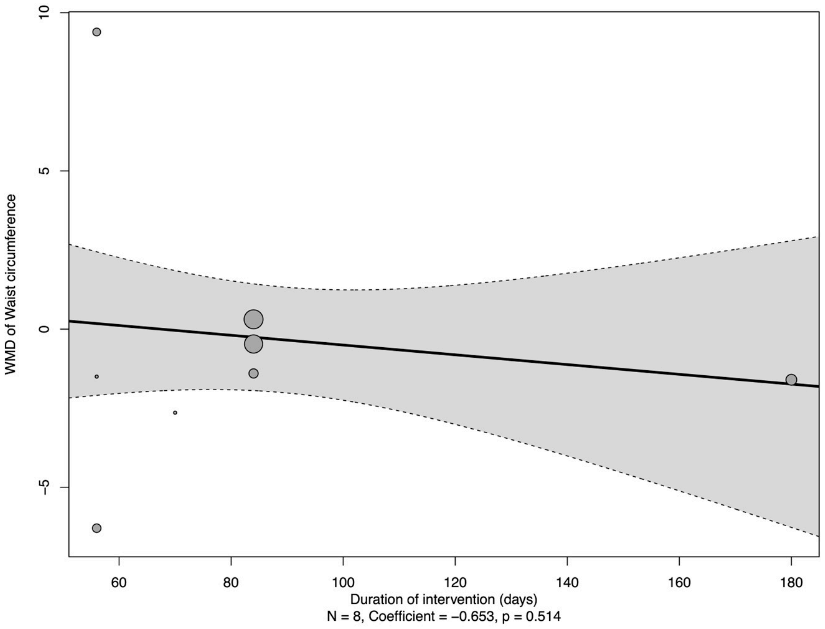

(c) SBP

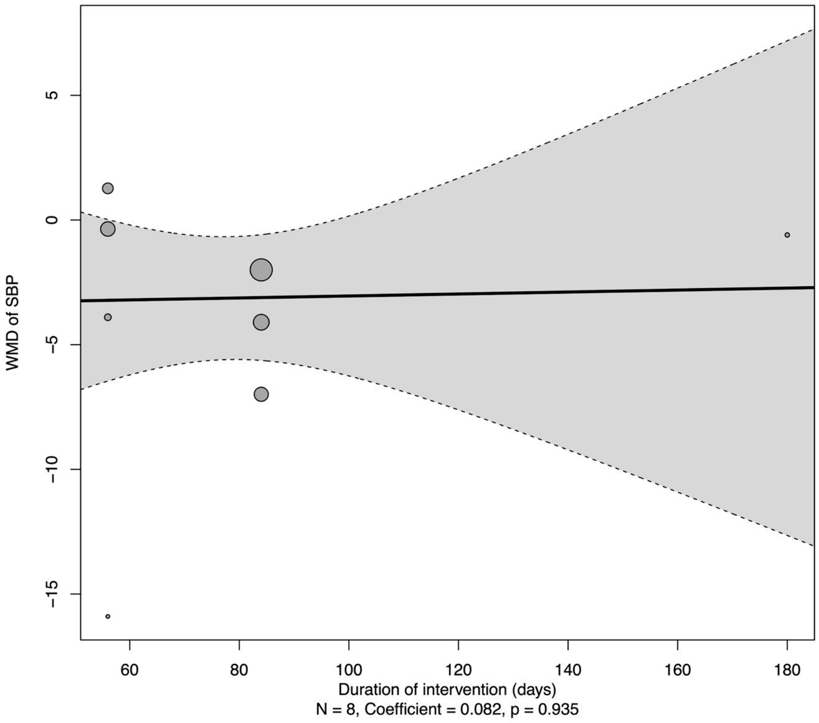

(d) DBP

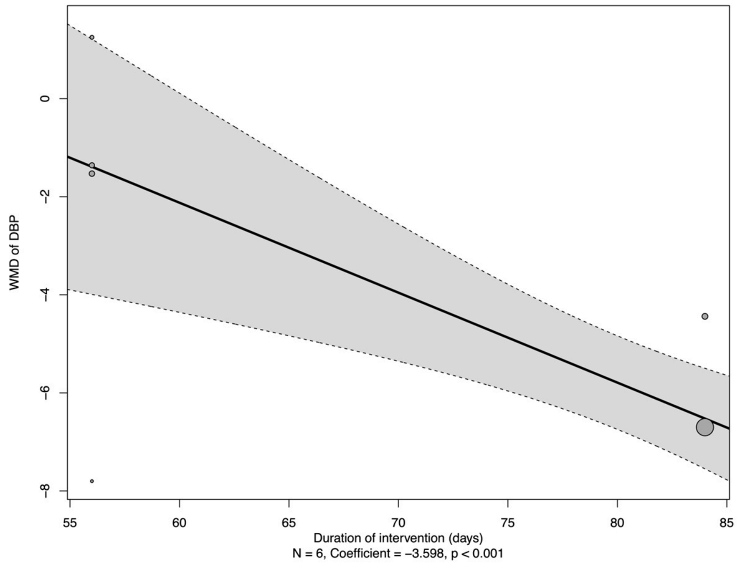

(e) FBG

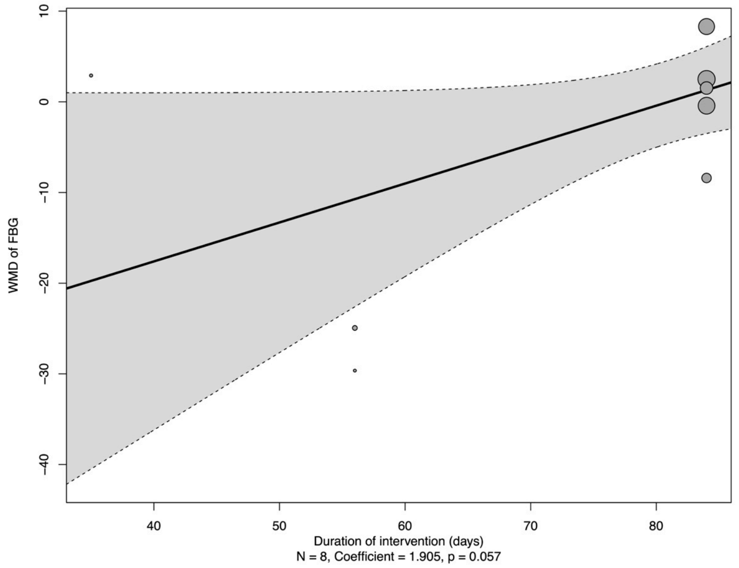

(f) HbA1C

-

(g) Insulin levels

-

(h) Triglycerides

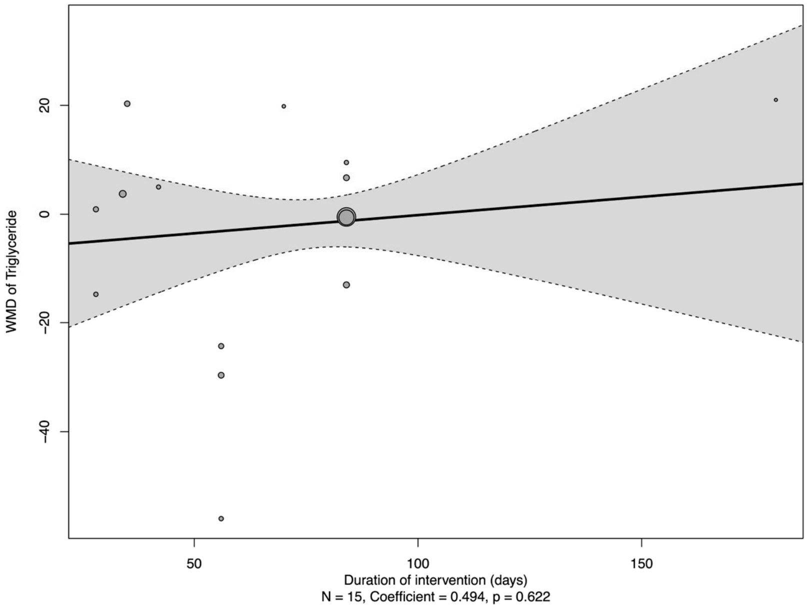

(i) Total cholesterol

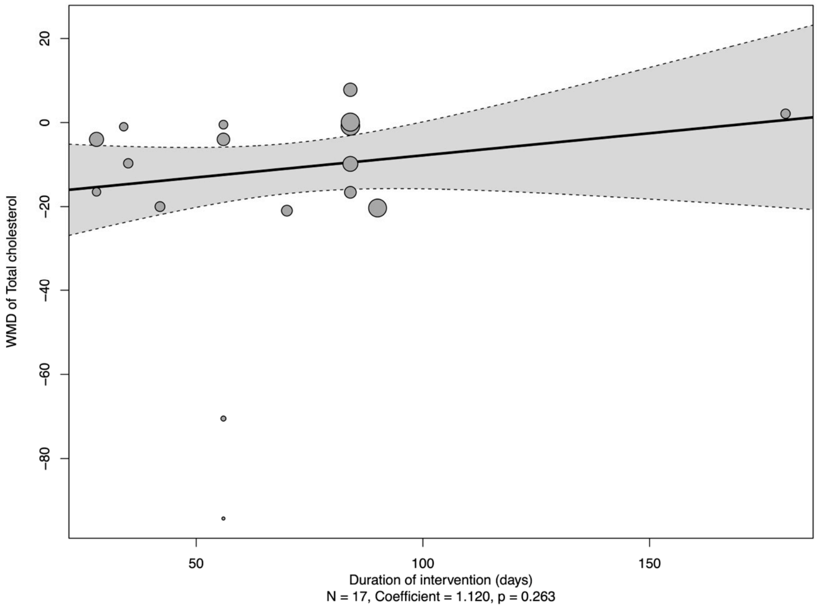

(j) LDL-C

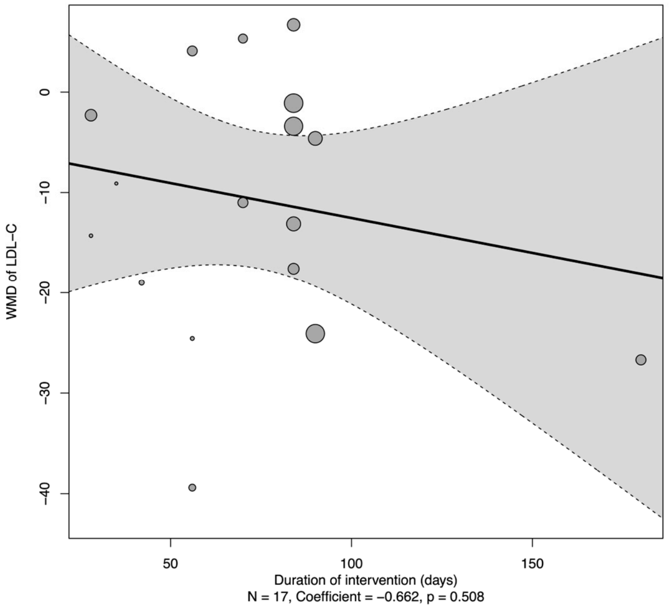
WMD of LDL−C
0
−10
−20
−30
−40
50
100
150
Duration of intervention (days)
N = 17, Coefficient = −0.662, p = 0.508

(k) HDL-C

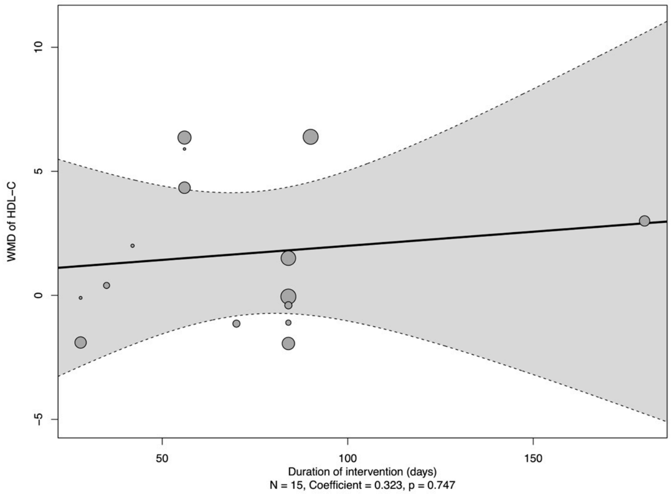
WMD of HDL−C
10
5
0
−5
50
100
150
Duration of intervention (days)
N = 15, Coefficient = 0.323, p = 0.747

Supplementary Material S14. Meta-regression analysis of the effect of rice bran supplementation dose (grams/day) on various metabolic parameters: (a) body mass index (BMI), (b) waist circumference, (c) systolic blood pressure (SBP), (d) diastolic blood pressure (DBP), (e) fasting blood glucose (FBG), (f) HbA1C, (g) insulin levels, (h) triglycerides, (i) total cholesterol, (j) low-density lipoprotein cholesterol (LDL-C), and (k) high-density lipoprotein cholesterol (HDL-C). The X-axis represents the rice bran dose, while the Y-axis represents the weighted mean difference (WMD) in each metabolic parameter. Each dot represents an individual study, with the regression line indicating the trend and the shaded area denoting the 95% confidence interval (CI).

(a) BMI

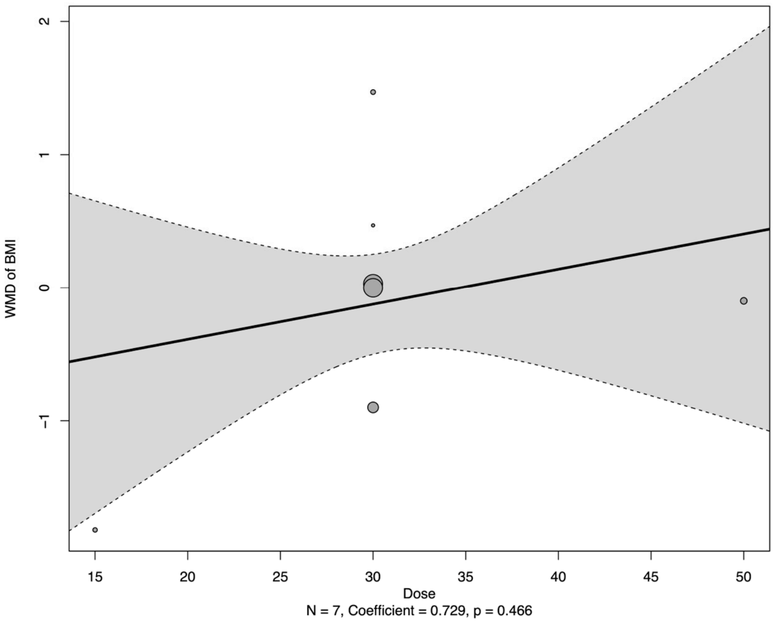

(b) Waist circumference

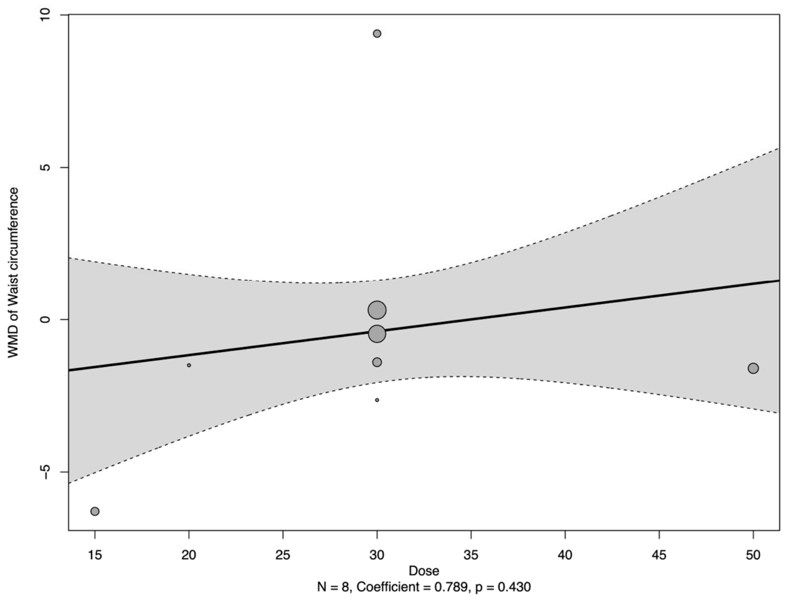

(c) SBP

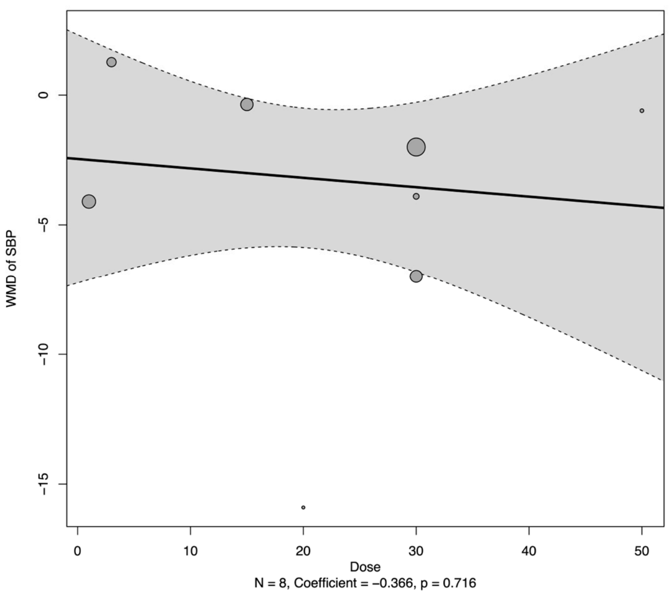

(d) DBP

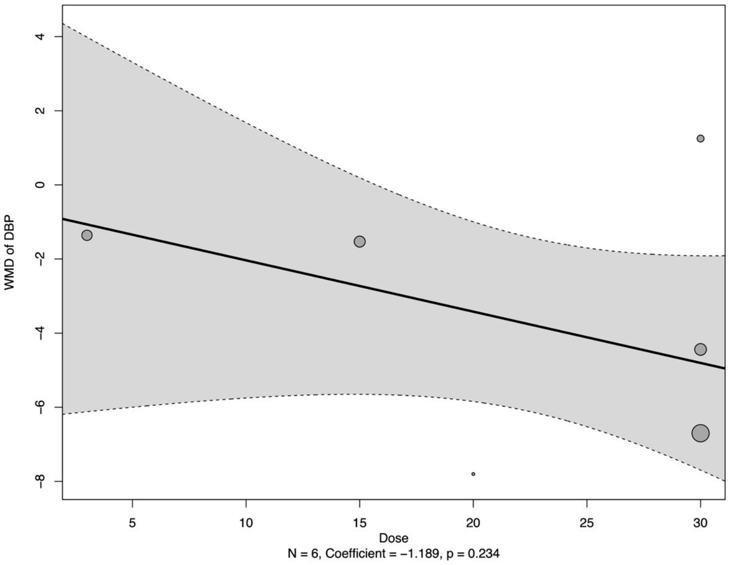

(e) FBG

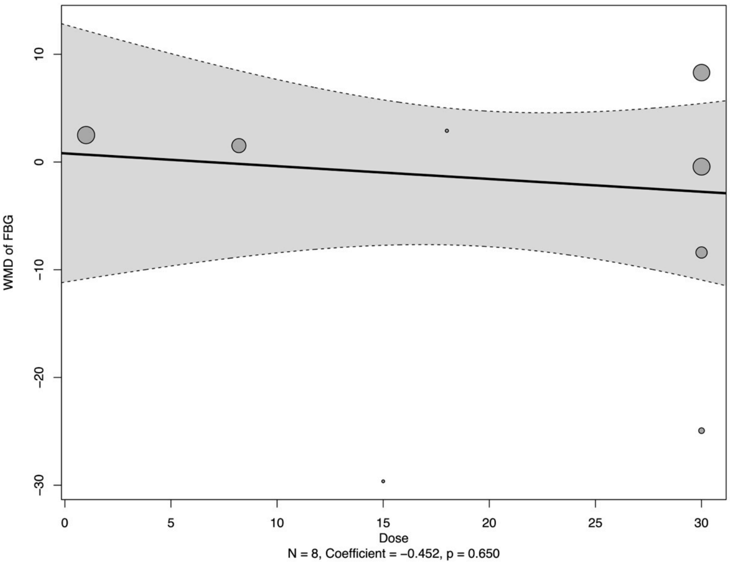

(f) HbA1C

-

(g) Insulin levels

-

(h) Triglycerides

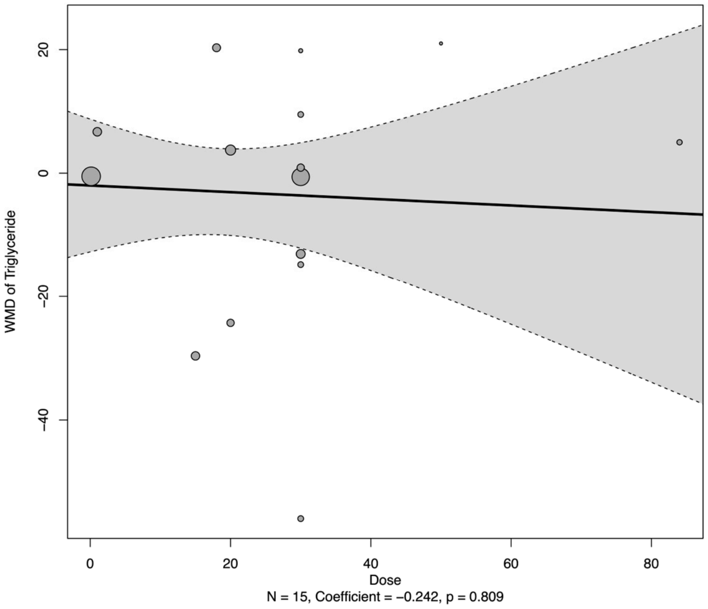

(i) Total cholesterol

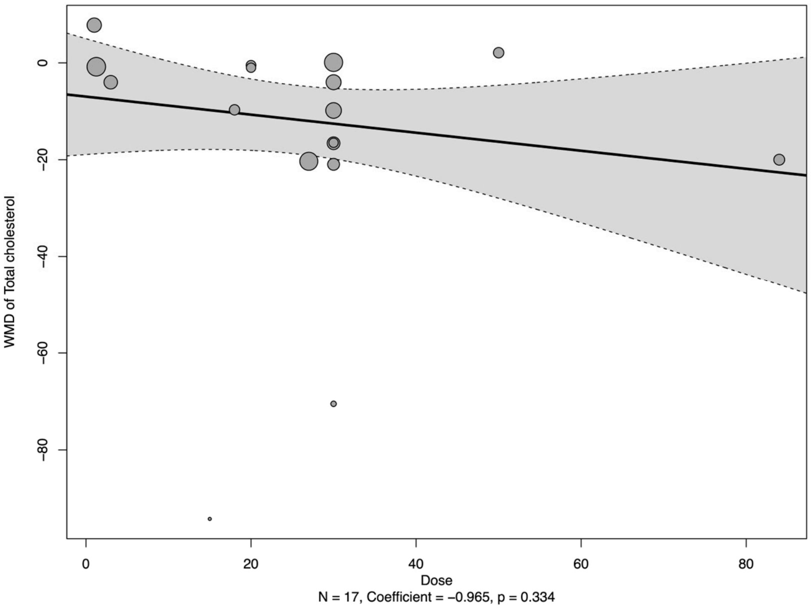

(j) LDL-C

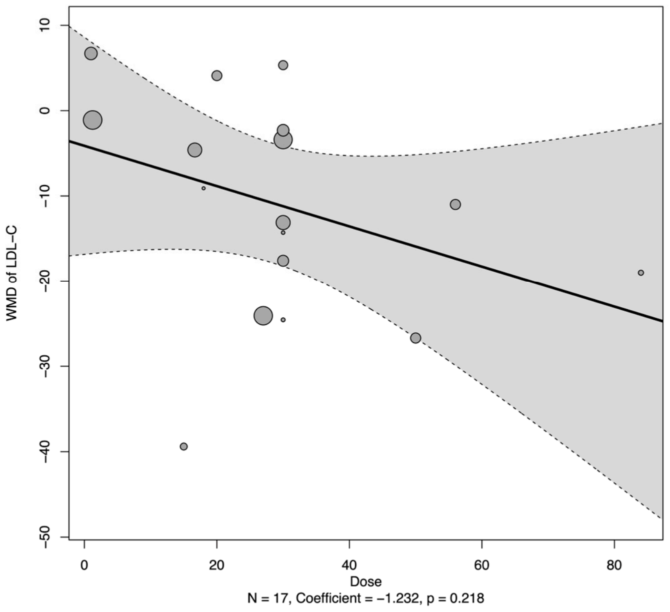

WMD of LDL−C
10
0
−10
−20
−30
−40
−50
0
20
40
60
80
Dose
N = 17, Coefficient = −1.232, p = 0.218

(k) HDL-C

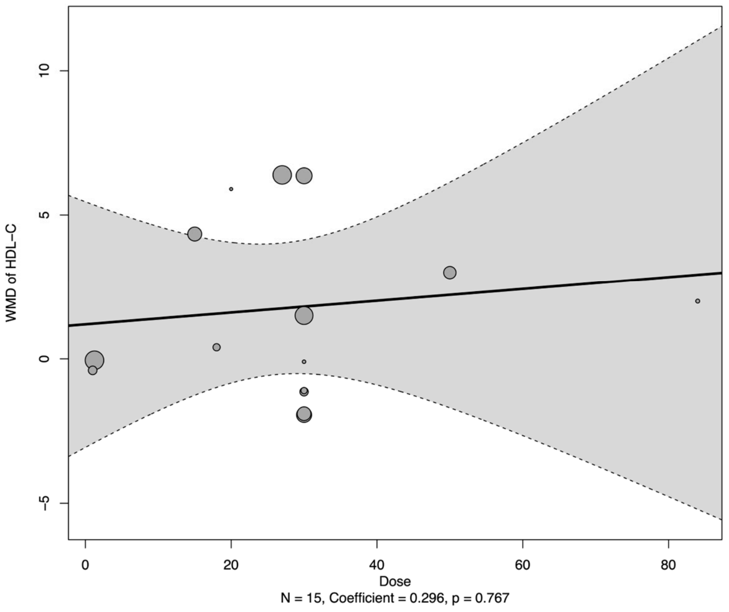

WMD of HDL−C
10
5
0
−5
0
20
40
60
80
Dose
N = 15, Coefficient = 0.296, p = 0.767

Supplementary Material S15. Subgroup analyses of rice bran supplementation: dose variation in rice bran or its bioactive compound supplementation and duration of intervention (days).

MetS-Related Parameters	Subgroups		Number of Studies	Effect Estimates	
				WMD	95% CI
Body mass index (BMI)	Overall		11	0.009	-0.413 to 0.432
	Dose variation in rice bran supplementation	≥ 20 g/d	6	-0.040	-0.320 to 0.250
		< 20 g/d	1	-1.820	-3.990 to 0.350
		Oryzanol	3	-0.490	-0.810 to -0.170
	Duration of intervention (days)	≥ 90	2	-0.520	-0.900 to -0.140
		< 90	9	-0.130	-0.320 to 0.590
Waist circumference	Overall		8	-0.332	-1.975 to 1.311
	Dose variation in rice bran supplementation	≥ 20 g/d	7	0.230	-1.280 to 1.730
		< 20 g/d	1	-6.290	-10.660 to -1.920
		Oryzanol	-	-	-
	Duration of intervention (days)	≥ 90	1	-1.600	-5.090 to 1.890
		< 90	7	-0.160	-1.950 to 1.640
Systolic blood pressure (SBP)	Overall		10	-3.336	-5.248 to -1.424
	Dose variation in rice bran supplementation	≥ 20 g/d	5	-4.820	-8.880 to -0.760
		< 20 g/d	3	-1.590	-4.730 to 1.550

		Oryzanol	2	-4.760	-8.250 to -1.270
	Duration of intervention (days)	≥ 90	1	-0.600	-11.100 to 9.900
		< 90	9	-3.470	-5.490 to -1.440
Diastolic blood pressure (DBP)	Overall		8	-3.145	-5.690 to -0.600
	Dose variation in rice bran supplementation	≥ 20 g/d	4	-4.840	-7.970 to -1.700
		< 20 g/d	2	-1.450	-4.400 to 1.500
		Oryzanol	2	-1.760	-4.580 to 1.070
	Duration of intervention (days)	≥ 90	-	-	-
		< 90	8	-3.150	-5.690 to -0.600
Fasting blood glucose (FBG)	Overall		10	-0.670	-4.844 to 3.505
	Dose variation in rice bran supplementation	≥ 20 g/d	4	-2.380	-11.380 to 6.610
		< 20 g/d	4	0.270	-6.750 to 7.280
		Oryzanol	2	-2.010	-6.070 to 2.060
	Duration of intervention (days)	≥ 90	-	-	-
		< 90	10	-0.670	-4.840 to 3.510
HbA1C	Overall		8	-0.199	-0.332 to -0.067
	Dose variation in rice bran supplementation	≥ 20 g/d	3	-0.250	-0.430 to -0.070
		< 20 g/d	2	-0.060	-0.700 to 0.260
		Oryzanol	3	-0.120	-0.320 to 0.070

	Duration of intervention (days)	≥ 90	1	-0.220	-0.370 to -0.070
		< 90	7	-0.190	-0.350 to -0.030
Insulin levels	Overall		5	-0.132	-1.098 to 0.834
	Dose variation in rice bran supplementation	≥ 20 g/d	2	-0.430	-1.510 to 0.660
		< 20 g/d	2	0.720	-1.690 to 3.130
		Oryzanol	1	2.440	-2.680 to 7.560
	Duration of intervention (days)	≥ 90	-	-	-
		< 90	5	-0.130	-1.100 to 0.830
Triglycerides	Overall		19	-7.570	-16.714 to 1.573
	Dose variation in rice bran supplementation	≥ 20 g/d	11	-2.650	-8.950 to 3.640
		< 20 g/d	4	-1.030	-15.780 to 13.710
		Oryzanol	4	-18.920	-29.790 to -8.050
	Duration of intervention (days)	≥ 90	2	-22.750	-24.860 to -20.640
		< 90	17	-3.750	-8.970 to 1.470
Total cholesterol	Overall		21	-13.594	-20.289 to -6.900
	Dose variation in rice bran supplementation	≥ 20 g/d	12	-12.720	-21.130 to -4.320
		< 20 g/d	5	-11.010	-26.330 to 4.320
		Oryzanol	4	-18.760	-39.150 to 1.630

	Duration of intervention (days)	≥ 90	2	-11.450	-20.890 to -6.750
		< 90	19	-13.820	-20.890 to -6.750
Low-density lipoprotein cholesterol (LDL-C)	Overall		22	-14.580	-21.124 to -8.036
	Dose variation in rice bran supplementation	≥ 20 g/d	12	-12.000	-20.420 to -3.590
		< 20 g/d	5	-7.230	-17.600 to 3.130
		Oryzanol	5	-27.770	-50.200 to -5.340
	Duration of intervention (days)	≥ 90	4	-22.930	-35.050 to -10.810
		< 90	18	-12.060	-17.460 to -6.670
High-density lipoprotein cholesterol (HDL-C)	Overall		20	3.074	0.829 to 5.319
	Dose variation in rice bran supplementation	≥ 20 g/d	11	1.980	-0.630 to 4.590
		< 20 g/d	4	2.650	-1.380 to 3.160
		Oryzanol	5	8.060	1.690 to 14.430
	Duration of intervention (days)	≥ 90	3	6.240	4.830 to 7.650
		< 90	17	2.360	0.510 to 4.210

Supplementary Material S16. Study characteristics included in the meta-analysis.

Author (Year)	Design	Country	Participants	Age (Year)	Sex	Intervention	Control	Number of Participants		Duration (Days)
								Intervention	Control	
Accinni (2006)	Randomized controlled trial	Italy	Dyslipidemic individuals	23–65	Both	Daily dietary supplementation with polyunsaturated fatty acids n-3 (660 mg eicosapentaenoic (EPA) and 440 mg docosahexaenoic (DHA)) and vitamin E (a-tocoferol, 4 mg) + oryzanol (40.2 mg) and niacin (18 mg)	Placebo (rice starch)	20	19	120
Balters (1981)	Randomized controlled trial	United States of America	Healthy individuals	20–50	Both	Basal diet with rice bran into the bread (20 g/d)	Placebo (basal diet)	10	10	34
Borresen (2016)	Randomized single-blind controlled trial	United States of America	Colorectal cancer patients	30–85	Both	Rice bran powder (30 g/d)	Placebo (rice starch)	9	10	28
Bumrungpert (2019)	Randomized controlled trial	Thailand	Hyperlipidemic individuals	20–60	Both	Rice bran oil (30 mL)	Oil without rice bran	15	15	28

Cheng (2010)	Randomized double-blind controlled trial	Taiwan	Type 2 diabetes patients	57–58	Both	Rice bran flour: 20 g of rice bran contained 3.9 g of oil	Placebo (milled rice flour)	17	11	84
Choi (2014)	Randomized double-blind controlled trial	Korea	Healthy individuals	25-70	Both	Rice bran fermented with Lentinus edodes (rice bran exo-biopolymer) in capsules (3 g/d)	Placebo (rice starch)	40	37	56
De Lellis (2024)	Randomized, double-blind controlled trial	Italy	Dyslipidemic individuals	18-70	Both	ROSSOPURO® Forte with Monacolins, γ-Aminobutyric Acid (GABA), and γ-Oryzanol (0.062 g/d)	Placebo	44	44	84
Gerhardt (1998)	Randomized controlled trial	United States of America	Hypercholesterolemic individuals	32-64	Both	A heat-stabilized, full-fat, medium-grain rice bran product (84 g/d)	Placebo (rice starch)	14	13	42
Ghorbani (2025)	Randomized open-label controlled trial	Iran	Metabolic syndrome individuals	30-65	Both	Standard diet with rice bran powder (15 g/d)	Standard diet without rice bran powder	24	19	56

Ito (2015)	Randomized controlled trial	Japan	Obese individuals with high LDL-C level	54	Males	Rice bran acylated steryl glucosides (0.05 g/d)	Placebo (rice starch)	40	20	84
Kim (2008)	Randomized double-blind controlled trial	Korea	Healthy overweight individuals	19–50	Females	Conjugated linoleic acid 3.0 g with oryzanol 0.3 g 6 capsules/d	Conjugate linoleic acid	14	12	84
Lai (2012)	Randomized single-blind controlled trial	Taiwan	Type 2 diabetes patients	56–57	Both	Rice bran oil-modified milk (18 g/d rice bran oil)	Placebo (rice starch)	18	17	35
Lin (2020)	Randomized controlled trial	Taiwan	Metabolic syndrome and healthy individuals	-	Both	Refined rice bran (20 g/d)	Refined oil without rice bran	26	17	56
Mahdavi-Roshan (2024)	Randomized open-label controlled trial	Iran	Metabolic syndrome individuals	45–70	Both	Standard diet with rice bran oil (30 g/d)	Standard diet without rice bran oil	23	21	56
Malve (2010)	Randomized double-blind controlled trial	India	Hyperlipidemic individuals	18–65	Both	Rice bran oil (16.67 g/d)	Blend oil without rice bran oil	35	31	90

Most (2005)	Randomized double-blind controlled trial	United States of America	Moderately hypercholesterolemic individuals	33	Both	Rice bran oil diet (56 g/d)	Control oil blend diet	14	14	70
Nhung (2016)	Randomized controlled trial	Vietnam	Post-menopausal with high LDL-C level (over 140 mg/dL)	45–65	Females	Pre-germinated brown Rice bran extract (50 g/d)	Placebo (rice starch)	30	30	180
Nikooyeh (2023)	Randomized double-blind controlled trial	Iran	Type 2 diabetes patients	20–65	Both	Oryzanol -fortified canola oil (30 g/d)	Unfortified canola oil (without oryzanol)	30	32	84
Ogawa (2019)	Randomized double-blind controlled trial	Japan	High-normal blood pressure individuals	45–64	Both	Processed rice bran (1 g/d)	Placebo (rice starch)	44	43	84
Ogawa (2018)	Randomized double-blind controlled trial	Japan	High-normal blood pressure and mild hypertension individuals	45–65	Both	Thermolysindigested rice bran (1 g/d)	Placebo	44	43	84

Qureshi (2001)	Randomized double-blind, cross-over controlled trial	United States of America	Hypercholesterolemic individuals	45	Both	Tocotrienol-rich fraction (0.2 g/d)	AHA Step-1 diet	18	18	35
Qureshi (2002)	Randomized controlled trial	United States of America	Hypercholesterolemic individuals	< 50	Both	Tocotrienol-rich fraction (0.2 g/d)	AHA Step-1 diet	18	18	35
Saphyakhajorn (2022)	Randomized controlled trial	Thailand	Overweight/ obese individuals with hypercholeste rolemia	18–60	Both	Defatted rice bran (30 g/d)	Placebo	30	31	84
Umin (2015)	Randomized single-blind controlled trial	Japan	Type 2 diabetes patients	35–60	Both	Rice bran and rice bran oil (8.2 g/d)	Placebo	12	11	84
Upadya (2015)	Randomized double-blind controlled trial	India	Hyperlipidemic individuals	25–45	Both	Blend oil with rice bran oil (1 L/person/month)	Blend oil without rice bran oil	39	41	90

Zavoshy (2012)	Randomized controlled trial	Iran	Hyperlipidemic individuals	25–65	Both	Low-calories diet with rice bran oil (30 g/d)	Low-calories diet without rice bran oil	14	36	70

AHA = American Heart Association; g = gram; d = day; L = liter; and mL = milliliter.
